# Kukoamine B, a Lycium-Derived Polyamine Alkaloid, Attenuates High-Fat-Diet-Induced Skeletal Muscle Dysfunction with the Involvement of LPS/TLR4/NF-κB-Mediated Metabolic Inflammation

**DOI:** 10.3390/biom16091275

**Published:** 2026-09-03

**Authors:** Shunling Yuan, Jiaxin Liu, Lihan Lin, Yiping Liu, Shan Xu, Liangwu Qiu

**Affiliations:** 1 Provincial University Key Laboratory of Sport and Health Science, School of Physical Education and Sport Sciences, Fujian Normal University, Fuzhou 350117, China; qbx20240038@yjs.fjnu.edu.cn (S.Y.); qbx20250041@yjs.fjnu.edu.cn (L.L.); ypliu1966@fjnu.edu.cn (Y.L.); 2 Department of Chemistry, Sungkyunkwan University, Suwon 16419, Republic of Korea; jxliu@g.skku.edu; 3 Fujian Key Laboratory of Innate Immune Biology, Biomedical Research Center of South China, College of Life Sciences, Fujian Normal University, Fuzhou 350117, China; 4 Department of Physical Education, Kunming Medical University, Kunming 650500, China

**Keywords:** kukoamine B, high-fat diet, Lycium, skeletal muscle dysfunction, metabolic inflammation, TLR4/NF-κB

## Abstract

A high-fat diet (HFD) promotes lipid metabolic disorders, chronic low-grade inflammation and skeletal muscle dysfunction. Kukoamine B (KB), a Lycium-derived polyamine alkaloid with reported anti-inflammatory and antioxidant activities, may protect against HFD-associated muscle impairment. C57BL/6J mice were fed an HFD and orally administered KB for 12 weeks. Muscle function was evaluated using inverted grid, forelimb grip strength and treadmill exhaustion tests. Histological, transcriptomic, ELISA, immunofluorescence and Western blot analyses were used to assess muscle injury and inflammatory signalling. KB reduced intramuscular lipid deposition, collagen accumulation and myofibre damage, improved muscle strength and exercise endurance, and partially restored AKT/mTOR-associated protein metabolic signalling. Transcriptomic analysis showed that KB downregulated HFD-activated inflammatory responses, chemokine signalling, lipopolysaccharide responses and Toll-like receptor-related pathways. KB also reduced plasma and skeletal muscle LPS levels, inhibited TLR4/MyD88/NF-κB signalling, and decreased TNF-α and IL-1β expression. TAK-242 mimicked the anti-inflammatory and muscle function-improving effects of KB, whereas their combination produced no statistically detectable additional effect under the conditions tested. These findings suggest that KB attenuates HFD-induced skeletal muscle dysfunction, potentially involving reduced LPS burden and suppression of TLR4/NF-κB-mediated metabolic inflammation.

## 1. Introduction

With rapid changes in global food systems and lifestyles, dietary patterns are shifting towards higher fat intake and greater energy density, accompanied by increasing trends in chronic metabolic diseases and the double burden of malnutrition [1,2]. Long-term consumption of a high-fat diet (HFD) not only causes lipid metabolic disorders and abnormal fat deposition but also induces chronic low-grade inflammation, oxidative stress and metabolic dysfunction across multiple tissues [3,4,5]. Compared with body weight gain alone, the effects of an HFD on functional status are more complex. Its adverse effects are reflected not only in dyslipidemia and fat accumulation but also in metabolic and inflammatory interactions among skeletal muscle, liver, intestine and the immune system [6,7]. Therefore, elucidating the effects of HFD on exercise capacity and metabolic homeostasis at the tissue and molecular levels, and identifying plant-derived pharmacological candidates, has important value for the management of metabolic inflammation-associated tissue dysfunction.

Skeletal muscle is the largest locomotor organ in the body and the main effector tissue responsible for physical activity, muscle strength and exercise endurance [8,9]. It is also a key metabolic organ that regulates glucose uptake, fatty acid oxidation and energy expenditure, thereby playing a central role in maintaining systemic glucose and lipid metabolic homeostasis [9,10]. Long-term HFD intake can induce ectopic lipid deposition, mitochondrial dysfunction, impaired insulin signalling and disordered protein metabolism in skeletal muscle, which may compromise myofibre structural integrity and contractile function [11,12]. Previous studies have shown that mice fed an HFD exhibit reduced muscle strength, decreased treadmill endurance and impaired exercise performance, accompanied by enhanced skeletal muscle inflammation, increased oxidative stress and disrupted proteostasis [11,13]. Thus, skeletal muscle is not only an important target tissue of HFD-induced metabolic abnormalities but also a key tissue basis for explaining HFD-related declines in exercise capacity.

HFD-induced chronic low-grade inflammation is considered an important mechanism linking nutritional excess, metabolic disorders and tissue dysfunction [14]. Under HFD conditions, impairment of the intestinal barrier and the entry of gut-derived lipopolysaccharide (LPS) into the circulation can lead to metabolic endotoxemia and further induce inflammatory responses in multiple tissues [15,16]. As a major component of the outer membrane of Gram-negative bacteria, LPS can be recognized by Toll-like receptor 4 (TLR4) and activate nuclear factor-kappa B (NF-κB) through the MyD88-dependent signalling pathway, thereby promoting the expression of pro-inflammatory cytokines such as TNF-α and IL-1β [17,18]. TLR4 is involved not only in pattern recognition and inflammatory responses in immune cells but also in metabolic regulation in skeletal muscle. Studies have shown that TLR4 can affect skeletal muscle substrate metabolism, and that its expression and signalling activation are closely associated with skeletal muscle inflammation under conditions of HFD feeding, lipid overload and insulin resistance [19,20,21]. Therefore, LPS/TLR4/NF-κB inflammatory signalling may represent an important molecular pathway through which an HFD affects skeletal muscle structure and exercise capacity.

Natural bioactive compounds have attracted increasing attention in phytopharmacological research because of their multi-target actions, low toxicity and potential therapeutic value. Kukoamine B (KB) is a polyamine alkaloid derived from Lycium species, including Lycii Cortex, and has anti-inflammatory, antioxidant and metabolic regulatory activities [22,23]. Previous studies have shown that KB can act as a dual inhibitor of LPS and CpG DNA and attenuate LPS-induced inflammatory responses by binding to LPS [24,25]. In LPS-induced inflammatory models, KB reduces plasma LPS levels, suppresses pro-inflammatory cytokine expression and alleviates inflammatory tissue injury [25]. In addition, KB improves insulin resistance, oxidative stress, inflammatory responses and dyslipidemia in rats with metabolic abnormalities induced by a high-fat/high-fructose diet [26]. These findings suggest that KB may have potential effects on HFD-related metabolic injury and inflammatory responses. However, direct evidence remains lacking as to whether KB can regulate HFD-induced changes in skeletal muscle structure, protein metabolism and exercise function, and whether these effects are associated with skeletal muscle LPS/TLR4/NF-κB inflammatory signalling.

Based on this background, the present study used an HFD-fed mouse model to systematically evaluate the effects of KB on blood lipid profiles, fat deposition, skeletal muscle morphology, protein metabolism and exercise capacity-related indicators. We further combined transcriptome sequencing, ELISA, immunofluorescence and Western blot analyses to examine the regulatory effects of KB on immune–inflammatory transcriptional features and the LPS/TLR4/NF-κB signalling pathway in skeletal muscle. In addition, the TLR4 inhibitor TAK-242 was used for pharmacological investigation to explore the role of TLR4/NF-κB inflammatory signalling in KB-mediated regulation of HFD-related changes in skeletal muscle function. TAK-242 is a selective inhibitor of TLR4 signalling that suppresses TLR4-mediated inflammatory signal transduction by interfering with the interaction between TLR4 and its downstream adaptor proteins [27]. This study aimed to provide experimental evidence supporting KB as a potential Lycium-derived phytochemical for the management of HFD-associated skeletal muscle structural injury and functional decline and to clarify whether its effects involve the suppression of LPS/TLR4/NF-κB-mediated metabolic inflammation in skeletal muscle.

## 2. Materials and Methods

### 2.1. Experimental Animals and Grouping

**Figure 1:**
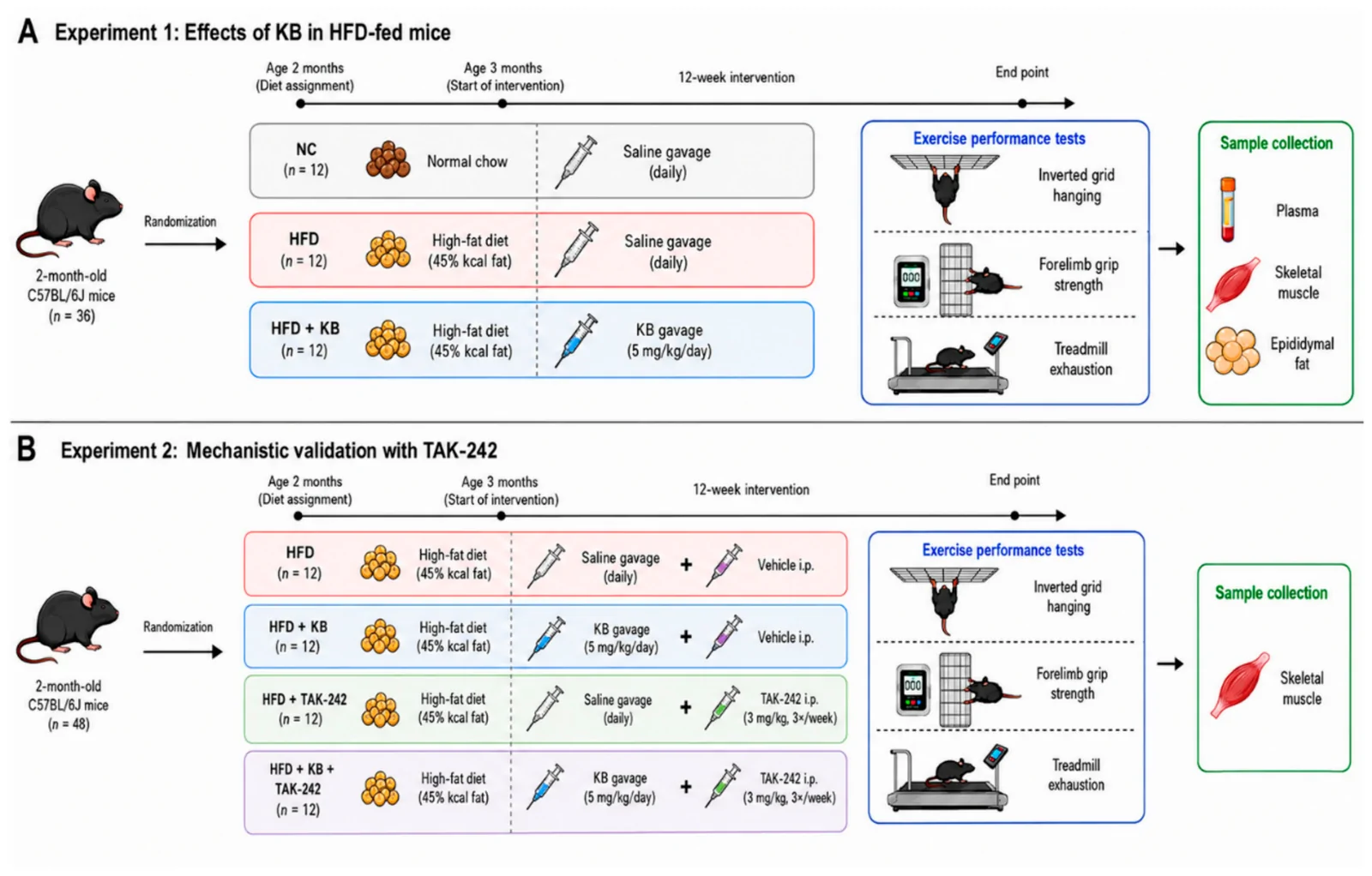
Experimental design. (**A**) Experiment 1: effects of KB on metabolic abnormalities, skeletal muscle function and exercise capacity in HFD-fed mice. (**B**) Experiment 2: pharmacological investigation using TAK-242.

Two-month-old male C57BL/6J mice were purchased from Changzhou Cavens Laboratory Animal Co., Ltd., Changzhou, China, (licence no. SCXK [Su] 2021-0001) and housed under specific pathogen-free conditions at four mice per cage, with a controlled temperature of 22 ± 2 °C and a 12 h light/dark cycle, with free access to food and water. In Experiment 1, 36 mice were randomly assigned to the normal chow group (NC, *n* = 12), HFD group (*n* = 12) or HFD plus KB group (HFD+KB, *n* = 12). Mice in the NC group were fed a standard chow diet, whereas mice in the HFD and HFD+KB groups were fed a high-fat diet providing 45% kcal from fat (Cat. No. Boaigang-12451M, Beijing Boaigang Biotechnology Co., Ltd., Beijing, China) from 2 months of age. The control diet and HFD were compositionally matched, with the primary difference being their fat content. From 3 months of age, mice in the HFD+KB group received KB by oral gavage for 12 weeks, whereas mice in the NC and HFD groups received an equal volume of normal saline by oral gavage. In Experiment 2, 48 mice were randomly assigned to the HFD, HFD+KB, HFD+TAK-242 or HFD+KB+TAK-242 groups, with 12 mice in each group. Mice in the HFD+KB and HFD+KB+TAK-242 groups received KB by oral gavage from 3 months of age. Mice in the HFD+TAK-242 and HFD+KB+TAK-242 groups received TAK-242 by intraperitoneal injection. The corresponding control groups received vehicle by oral gavage or intraperitoneal injection. All interventions lasted for 12 weeks. After the intervention period, exercise capacity tests were performed, and plasma, skeletal muscle and epididymal fat samples were collected for subsequent biochemical, histological and molecular analyses (Figure 1). No formal a priori sample size calculation was performed; group sizes were determined based on previous related animal studies and the experimental design. Samples used for the different assays were derived from the same experimental cohort. Because different assays required different sample types and tissue-processing procedures, the number of animals included in individual analyses varied, with the exact sample size specified in the corresponding figure legends.

### 2.2. KB Administration

KB was purchased from Shanghai Yuanye Bio-Technology Co., Ltd. (Cat. No. B20291, Shanghai, China) and stored at 2–8 °C protected from light according to the manufacturer’s instructions. KB was dissolved in normal saline and administered by oral gavage at a dose of 5 mg/kg/day at a volume of 10 mL/kg body weight, with the administration volume adjusted according to changes in body weight. The dosing solution was freshly prepared before administration. The purity of KB was 98% as stated in the certificate of analysis provided by the manufacturer, and its chemical identity was reported to be confirmed by HPLC/LC–MS according to the supplier’s quality control report. The chemical identity and purity of KB were not independently verified in the present study. The dose of 5 mg/kg/day was selected based on a previous in vivo study in which KB at 5 mg/kg effectively attenuated obesity and associated metabolic abnormalities [28]. Given the demonstrated efficacy of this dose in an obesity-related metabolic context, 5 mg/kg/day was selected to evaluate the effects of KB on HFD-induced metabolic inflammation and skeletal muscle dysfunction in the present study. KB intervention began when the mice were 3 months old and continued for 12 weeks, with gavage performed at a fixed time each day. In Experiment 1, mice in the HFD+KB group received KB by oral gavage, whereas mice in the NC and HFD groups received an equal volume of normal saline. In Experiment 2, mice in the HFD+KB and HFD+KB+TAK-242 groups received KB by oral gavage, whereas mice in the HFD and HFD+TAK-242 groups received an equal volume of normal saline.

### 2.3. TAK-242 Administration

TAK-242 was purchased from Shanghai Yuanye Bio-Technology Co., Ltd. (Cat. No. S80562, Shanghai, China) and administered by intraperitoneal injection from 3 months of age. TAK-242 was initially dissolved in DMSO to prepare a 50 mg/mL stock solution and subsequently diluted with normal saline to a working concentration of 0.5 mg/mL, resulting in a final vehicle containing 1% DMSO in normal saline. TAK-242 was administered three times per week at a dose of 3 mg/kg. The injection volume was 6 mL/kg body weight and was adjusted as body weight changed [29]. In Experiment 2, mice in the HFD+TAK-242 and HFD+KB+TAK-242 groups received TAK-242 by intraperitoneal injection, whereas mice in the HFD and HFD+KB groups received an equal volume of vehicle (1% DMSO in normal saline) by intraperitoneal injection.

### 2.4. Exercise Capacity Tests

Five mice were randomly selected from the 12 mice in each group for exercise capacity testing before the functional assessments were performed, independently of their subsequent performance. The same five mice underwent all three functional tests. The tests were performed in a fixed order on three consecutive days: the forelimb grip strength test on Day 1, the inverted grid test on Day 2, and the treadmill exhaustion test on Day 3. Formal investigator blinding was not implemented during the functional assessments; however, the same predefined testing procedures and endpoint criteria were applied consistently across all experimental groups.

#### 2.4.1. Forelimb Grip Strength Test

Forelimb grip strength was measured using a digital grip strength metre. Before testing, each mouse was gently removed from its cage and positioned along the body axis so that its forelimbs contacted the metal grid of the grip strength metre. After the mouse grasped the grid spontaneously, the tail was slowly pulled backwards to generate forelimb traction force. The instantaneous maximal grip force was recorded on the digital display. Each mouse was measured three times, with a 2 min interval between measurements, and the mean value was used as the final forelimb grip strength.

#### 2.4.2. Inverted Grid Test

Each mouse was placed on a metal wire grid and allowed to grasp the grid firmly with all four limbs. The grid was then slowly rotated by 180° so that the mouse was suspended in a supine position beneath the grid. Timing began once the grid was fully inverted, and the latency to fall was recorded. Each mouse was tested three times, with a 10 min interval between trials, and the mean value was used as the final result. If a mouse did not fall within 300 s, then the latency was recorded as 300 s. A soft pad was placed beneath the grid during the test to prevent injury when the mouse fell.

#### 2.4.3. Treadmill Exhaustion Test

The treadmill exhaustion test was performed using a small-animal treadmill according to previously described incremental-load protocols, with minor modifications [30,31]. No separate treadmill-acclimatization programme was performed before the formal exhaustion test. The treadmill incline was set at 0°. At the beginning of the formal test, mice ran at 10 m/min for 5 min as a warm-up. The running speed was then increased to 12 m/min and subsequently increased by 2 m/min every 3 min until the mice reached exhaustion. Exhaustion was defined as the inability to maintain running and resume running despite repeated gentle tactile encouragement. The total time to exhaustion, cumulative running distance and running speed at exhaustion were recorded during the test.

### 2.5. Biochemical Assays

Following the intervention, mice were fasted for 12 h, and blood samples were collected into anticoagulant tubes. Plasma was separated by centrifugation at 3000× *g* for 10 min at 4 °C. TC and TG levels were detected using commercial biochemical kits (Ruixin Biotech, Cat. Nos. RXWB0294 and RX634, Quanzhou, China) according to the manufacturer’s instructions. Absorbance was read with a microplate reader, and concentrations were calculated based on standard curves.

### 2.6. Histological Analysis of Skeletal Muscle Sections

Quadriceps tissues were collected from the mid-belly region of the muscle and fixed in 4% paraformaldehyde for 24 h. After routine dehydration, clearing and paraffin embedding, serial sections with a thickness of 4 μm were prepared. For Oil Red O staining, skeletal muscle samples were embedded in OCT compound and cut into frozen sections with a thickness of 6–10 μm. For histological quantification, three non-consecutive sections per animal were analyzed, with five randomly selected non-overlapping fields evaluated per section.

#### 2.6.1. Haematoxylin and Eosin Staining

Paraffin sections were deparaffinized in xylene and rehydrated through a graded ethanol series. The sections were then stained sequentially with haematoxylin and eosin, followed by dehydration, clearing and mounting with neutral resin. After staining, skeletal muscle fibre arrangement, myofibre morphology and tissue structural integrity were observed under a light microscope. For H&E-stained sections, skeletal muscle area was quantified using ImageJ (version 1.53t, NIH, Bethesda, MD, USA) and expressed as the percentage of the total analyzed field occupied by skeletal muscle fibres (skeletal muscle area, %).

#### 2.6.2. WGA Staining

After routine dewaxing, hydration, antigen retrieval and blocking, skeletal muscle sections were incubated with fluorescent WGA solution in the dark to visualize the sarcolemmal boundaries. Nuclei were stained with DAPI, and the sections were mounted with anti-fade medium. Images were obtained using a fluorescence microscope to evaluate myofibre cross-sectional profiles.

#### 2.6.3. Sirius Red Staining

Following routine dewaxing and hydration, paraffin sections were stained with Sirius Red solution. The sections were then rinsed, dehydrated, cleared and mounted with neutral resin. Collagen fibre deposition was observed under a light microscope, and the collagen-positive area was quantified from the acquired images.

#### 2.6.4. Oil Red O Staining

Frozen sections were equilibrated at room temperature, fixed, washed and stained with Oil Red O working solution. After differentiation and haematoxylin counterstaining, the sections were mounted with an aqueous mounting medium. Lipid droplets in skeletal muscle were observed under a light microscope. Images were obtained under consistent acquisition conditions, and multiple randomly selected non-overlapping fields from each mouse were analyzed.

### 2.7. Western Blot Assay

Skeletal muscle tissues were cut into small pieces and mechanically homogenized in ice-cold RIPA buffer containing protease and phosphatase inhibitors to obtain whole-tissue protein lysates. The lysates were centrifuged at 12,000× *g* for 15 min at 4 °C, and the supernatants were collected. Protein concentration was determined with a BCA protein assay kit (Servicebio, Cat. No. G2007, Wuhan, China). Equal amounts of protein were denatured, separated by SDS-PAGE using a RealBand Three-Color Prestained Protein Marker (10–180 kDa; Sangon Biotech, Cat. No. C610013, Shanghai, China) as the molecular weight standard and transferred onto PVDF membranes.

After blocking with 5% non-fat milk or BSA for 1 h, the membranes were incubated with the indicated primary antibodies overnight at 4 °C, followed by HRP-conjugated secondary antibodies for 1 h at room temperature. Protein signals were developed using enhanced chemiluminescence and captured with a gel imaging system. Band density was measured using ImageJ. Phosphorylated proteins were normalized to their respective total proteins, including p-AKT/AKT, p-mTOR/mTOR and p-p65/p65, while other proteins were normalized to α-tubulin.

All primary antibodies used in this study were purchased from Proteintech Group (Wuhan, China). The antibodies were as follows: p-AKT (Cat. No. 66444-1-Ig, 1:2000), AKT (Cat. No. 10176-2-AP, 1:2000), p-mTOR (Cat. No. 80596-1-RR, 1:5000), mTOR (Cat. No. 28273-1-AP, 1:2000), FBXO32 (Cat. No. 67172-1-Ig, 1:15,000), MuRF-1 (Cat. No. 55456-1-AP, 1:1000), TLR4 (Cat. No. 19811-1-AP, 1:500), MyD88 (Cat. No. 23230-1-AP, 1:5000), phosphorylated NF-κB p65 (p-p65) (Cat. No. 82335-1-RR, 1:2000), NF-κB p65 (p65) (Cat. No. 80979-1-RR, 1:5000) and α-tubulin (Cat. No. 80762-1-RR, 1:5000).

### 2.8. RNA Sequencing

Total RNA was extracted from skeletal muscle tissues using TRIzol reagent (Invitrogen, Carlsbad, CA, USA). RNA quality and integrity were assessed before library preparation. RNA-seq libraries were constructed and sequenced on an Illumina platform. After removing adapter sequences and low-quality reads, clean reads were aligned to the mouse reference genome GRCm39 (Mus musculus) using HISAT2 (version 2.2.1). Gene-level read counts were quantified using featureCounts (Subread version 2.0.3) based on the corresponding GENCODE mouse gene annotation (release M31). Differential expression analysis was performed using DESeq2 (version 1.38.3). Genes with |log2 fold change| > 1 and a nominal *p*-value < 0.05 were considered differentially expressed genes (DEGs). Multiple-testing-adjusted q-values were also obtained from the differential expression analysis and are provided in the complete differential expression tables in the Supplementary Materials.

Principal component analysis (PCA) and sample-to-sample correlation analysis were used to assess sample clustering and identify potential outliers. Potential batch effects were evaluated based on sample metadata and PCA clustering. No samples were excluded as outliers. GO and KEGG pathway enrichment analyses were performed using clusterProfiler (version 4.6.2), and enrichment significance was evaluated using Benjamini–Hochberg-adjusted *p*-values. Gene set enrichment analysis (GSEA) was performed using GSEA software (version 4.3.2) based on Gene Ontology (GO) and Kyoto Encyclopedia of Genes and Genomes (KEGG) pathway gene sets for Mus musculus. Normalized enrichment scores (NES) and FDR q-values were used to evaluate the magnitude and statistical significance of gene set enrichment.

### 2.9. ELISA Detection

LPS levels in plasma and skeletal muscle were determined using a commercial ELISA kit (Ruixin Biotech, Cat. No. JRXW202535, Quanzhou, China) according to the manufacturer’s instructions. Blood samples were collected using sterile disposable materials and transferred into sterile tubes. During sample preparation, clean disposable pipette tips and tubes were used to minimize potential exogenous endotoxin contamination. Skeletal muscle tissues were weighed and homogenized in ice-cold phosphate-buffered saline (PBS) at a tissue-to-buffer ratio of 1:9 (*w*/*v*). The homogenates were centrifuged at 12,000× *g* for 10 min at 4 °C, and the supernatants were collected for analysis. Plasma and muscle homogenate supernatants were diluted as required to ensure that the measured concentrations fell within the linear range of the standard curve. Absorbance was measured at 450 nm, and LPS concentrations were calculated from the standard curve. Skeletal muscle LPS levels were normalized to the total protein concentration of the corresponding tissue homogenate and expressed as LPS per unit of protein. Samples from different experimental groups were processed under identical conditions and measured in parallel on the same assay plate whenever possible to minimize analytical variation.

### 2.10. Immunofluorescence

Skeletal muscle paraffin sections were subjected to routine dewaxing, gradient ethanol hydration and citrate-mediated antigen retrieval. After blocking non-specific binding, the sections were incubated with primary antibodies against CD68, TLR4, IL-1β and TNF-α overnight at 4 °C. The next day, sections were washed and treated with corresponding fluorescence-labelled secondary antibodies in the dark. DAPI was used for nuclear counterstaining, and the slides were mounted with anti-fade medium. Images were obtained with a fluorescence or confocal microscope under consistent acquisition settings. ImageJ was used to quantify fluorescence signals from randomly selected non-overlapping fields, and the results were expressed as mean fluorescence intensity or positive staining area. For each marker, the threshold was set to distinguish specific fluorescence signals from background fluorescence and, once determined, was kept constant for all images of the same marker across experimental groups. The same analysis parameters were applied to all groups without group-specific or image-specific threshold adjustment.

### 2.11. Statistical Analysis

All data were analyzed and plotted using GraphPad Prism 9.5 and are shown as the mean ± standard deviation. Normality and homogeneity of variance were examined before statistical testing. For normally distributed data with equal variance, intergroup differences were analyzed by one-way ANOVA with Bonferroni post hoc correction. For Experiment 2, two-way ANOVA was additionally performed with KB treatment (absent/present) and TAK-242 treatment (absent/present) as fixed factors to evaluate the main effects of KB and TAK-242 and the KB × TAK-242 interaction. *p* < 0.05 was considered statistically significant, with significance indicated as *p* < 0.05 or *p* < 0.01 in the figures.

## 3. Results

### 3.1. Kukoamine B Alleviates HFD-Induced Dyslipidemia and Exercise Capacity Decline

**Figure 2:**
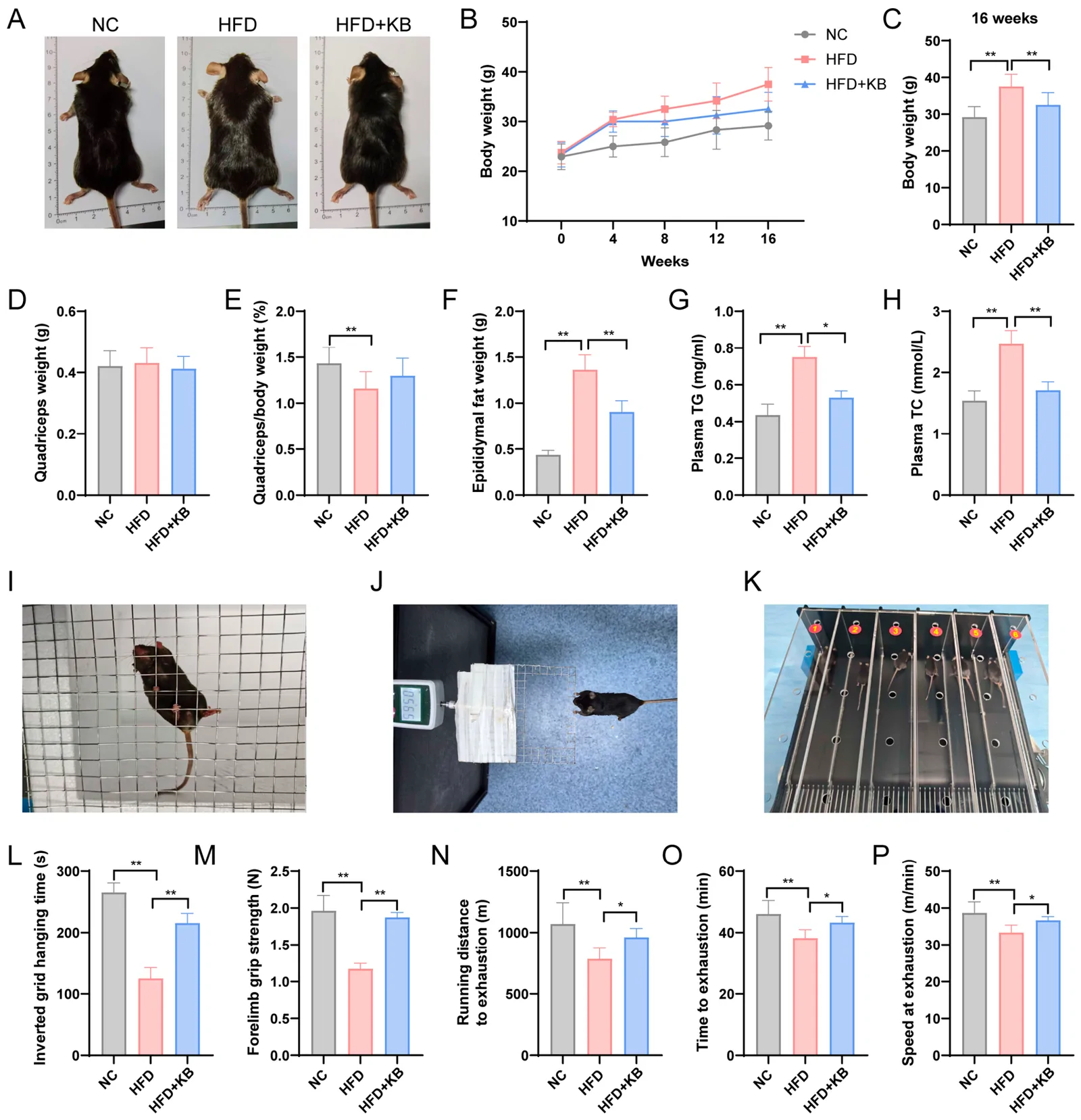
Kukoamine B improves HFD-induced metabolic disorders and exercise capacity decline in mice. (**A**) Representative whole-body appearance of mice in each group at the end of the experiment. (**B**) Body weight changes during the 16-week intervention period. (**C**) Final body weight at week 16. (**D**) Quadriceps weight. (**E**) Quadriceps weight/body weight ratio. (**F**) Epididymal fat weight. (**G**,**H**) Plasma TG and TC levels. (**I**–**K**) Representative images of the inverted grid test, forelimb grip strength test and treadmill exhaustion test. (**L**) Inverted grid hanging time. (**M**) Forelimb grip strength. (**N**) Running distance to exhaustion. (**O**) Time to exhaustion. (**P**) Running speed at exhaustion. Longitudinal body weight data in panel (**B**) are presented descriptively to illustrate temporal trends, whereas statistical comparisons of body weight were based on the final body weight measurements shown in panel (**C**). Data are presented as the mean ± SD. For panels (**B**–**H**), *n* = 12 mice per group; for panels (**L**–**P**), *n* = 5 mice per group. * *p* < 0.05, ** *p* < 0.01.

At the end of the experiment, mice in the HFD group showed an enlarged body size compared with those in the NC group. Body weight showed a progressive increase during the experimental period, with the HFD+KB group exhibiting a lower overall trend than the HFD group (Figure 2B). At week 16, the final body weight was significantly higher in the HFD group than in the NC group and significantly lower in the HFD+KB group than in the HFD group (Figure 2C). No significant difference in absolute quadriceps weight was observed among the groups (Figure 2D). However, the quadriceps weight/body weight ratio was significantly lower in the HFD group than in the NC group, whereas this ratio was partially restored after KB intervention (Figure 2E). HFD also significantly increased epididymal fat weight and elevated plasma TG and TC levels, while KB intervention markedly reduced epididymal fat accumulation and plasma TG and TC levels (Figure 2F–H). These results indicate that KB alleviates HFD-induced body weight gain, fat accumulation and lipid metabolic abnormalities.

The inverted grid test, forelimb grip strength test and treadmill exhaustion test were further used to evaluate skeletal muscle function and overall exercise capacity in mice (Figure 2I–K). The HFD group showed significant reductions in inverted grid hanging time, forelimb grip strength, running distance to exhaustion, time to exhaustion and running speed at exhaustion. After KB intervention, all these exercise capacity-related indicators were markedly improved (Figure 2L–P). These results suggest that KB improves muscle strength- and exercise capacity-related outcomes in HFD-fed mice.

### 3.2. Kukoamine B Improves HFD-Induced Skeletal Muscle Morphological Damage and Abnormal Protein Metabolism

**Figure 3:**
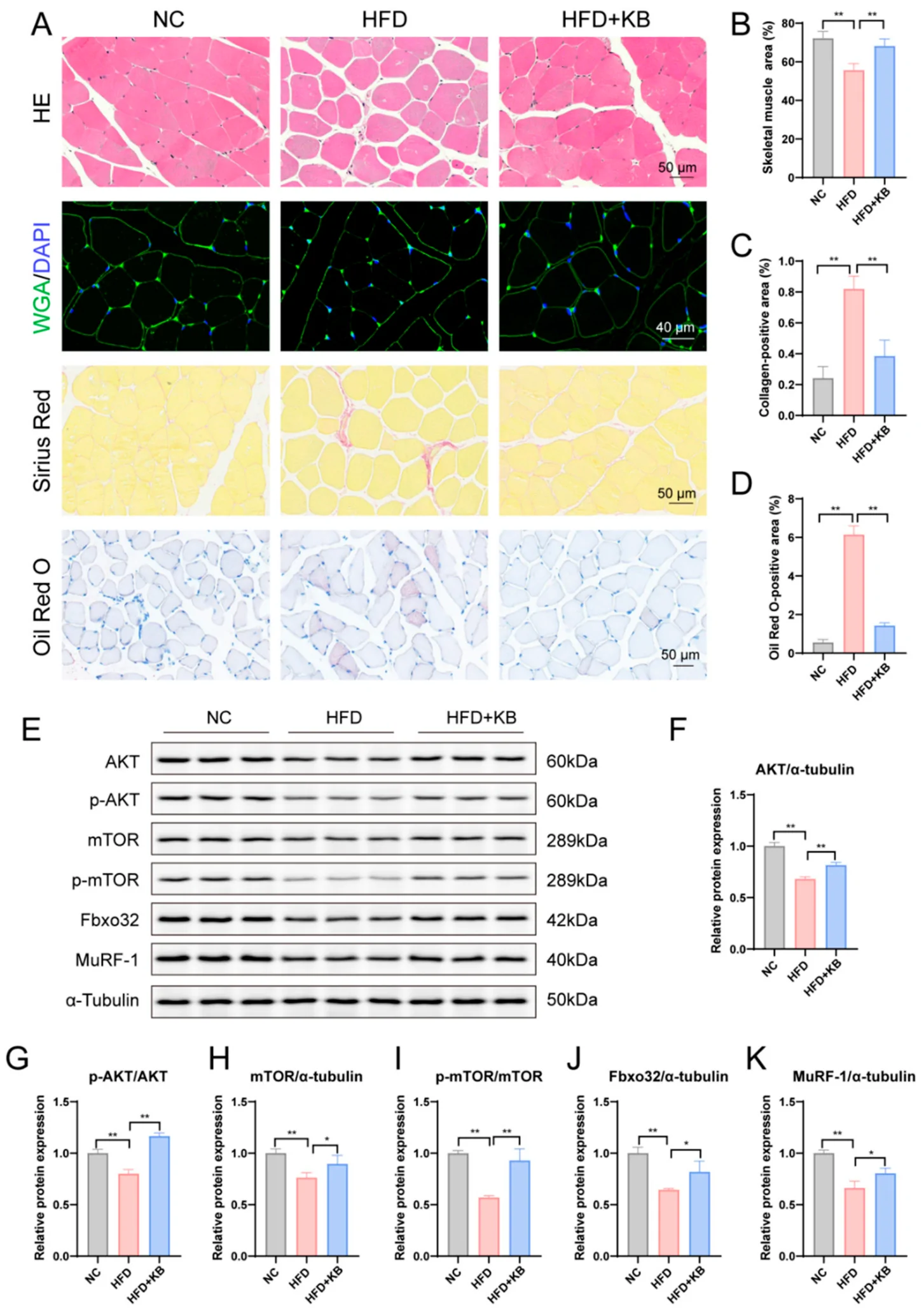
Kukoamine B improves HFD-induced skeletal muscle morphological damage and abnormal protein metabolism. (**A**) Representative images of haematoxylin and eosin staining, WGA/DAPI immunofluorescence staining, Sirius Red staining and Oil Red O staining in skeletal muscle from each group. (**B**) Quantitative analysis of skeletal muscle area in H&E-stained sections, expressed as the percentage of the total analyzed field occupied by skeletal muscle fibres. (**C**) Quantitative analysis of the Sirius Red-positive collagen area. (**D**) Quantitative analysis of the Oil Red O-positive area. (**E**) Representative Western blot images of AKT, p-AKT, mTOR, p-mTOR, FBXO32, MuRF-1 and α-tubulin protein expression in skeletal muscle. (**F**) AKT/α-tubulin protein expression. (**G**) p-AKT/AKT ratio. (**H**) mTOR/α-tubulin protein expression. (**I**) p-mTOR/mTOR ratio. (**J**) FBXO32/α-tubulin protein expression. (**K**) MuRF-1/α-tubulin protein expression. Data are presented as the mean ± SD. For panels (**A**–**D**), *n* = 4 per group; for panels (**E**–**K**), *n* = 5 per group. * *p* < 0.05, ** *p* < 0.01. Original Western blot images are provided in the Supplementary Materials.

To investigate whether the improvement in HFD-induced exercise capacity decline following KB treatment was accompanied by changes in skeletal muscle structure and protein metabolism-related signalling, skeletal muscle morphology, collagen deposition, lipid deposition and protein metabolism-related proteins were examined. Haematoxylin and eosin staining showed that skeletal muscle fibres in the HFD group were loosely arranged, with widened interstitial spaces and a significantly reduced skeletal muscle area compared with the NC group. In contrast, KB intervention improved muscle fibre arrangement and significantly increased the skeletal muscle area compared with the HFD group (Figure 3A,B). WGA/DAPI staining further showed that KB improved myofibre morphology (Figure 3A). Sirius Red staining showed that HFD significantly increased collagen deposition in skeletal muscle, whereas KB intervention significantly reduced the collagen-positive area (Figure 3A,C). Oil Red O staining showed that lipid deposition was markedly increased in the HFD group, whereas KB intervention significantly reduced lipid accumulation in skeletal muscle (Figure 3A,D). Western blot analysis showed that HFD reduced total AKT and mTOR protein levels and the p-AKT/AKT and p-mTOR/mTOR ratios, whereas KB intervention significantly increased these parameters (Figure 3E–H). FBXO32 and MuRF-1 protein expression was also decreased in the HFD group and increased following KB intervention (Figure 3I–K). Together, these findings indicate that KB ameliorates HFD-associated skeletal muscle structural abnormalities, collagen deposition and lipid accumulation and is accompanied by alterations in AKT/mTOR-related signalling and the expression of the protein metabolism-related factors FBXO32 and MuRF-1.

### 3.3. HFD Induces Immune–Inflammatory Activation and Suppresses PI3K-Related Metabolic Regulatory Signalling in Skeletal Muscle

**Figure 4:**
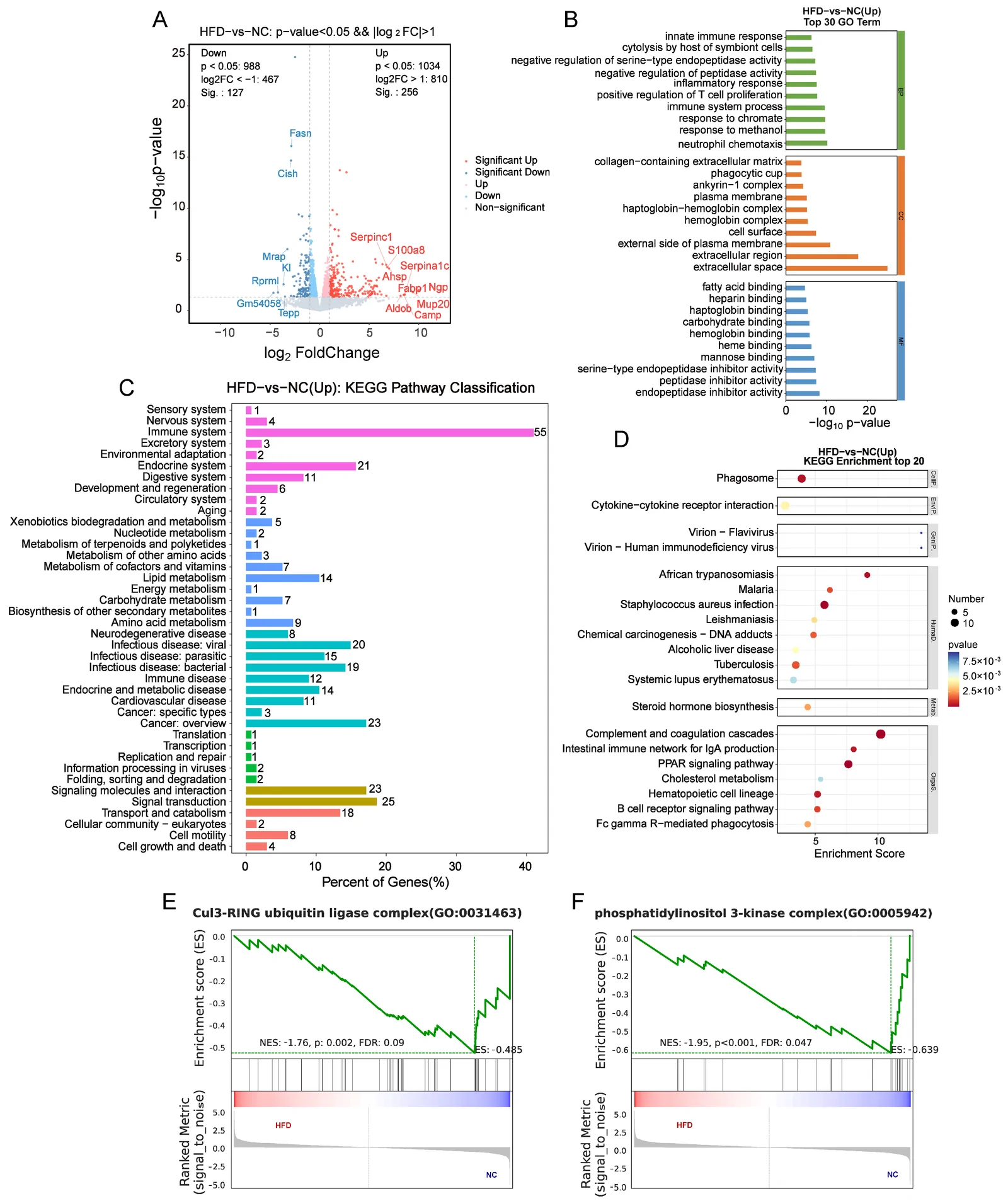
HFD induces immune inflammation-related transcriptional changes and suppresses PI3K-related signalling in skeletal muscle. (**A**) Volcano plot of differentially expressed genes in skeletal muscle between the NC and HFD groups. (**B**) GO functional enrichment analysis of significantly upregulated genes in the HFD group compared with the NC group. (**C**) KEGG pathway classification analysis of upregulated differentially expressed genes in the HFD group. (**D**) Bubble plot of KEGG enrichment analysis of upregulated differentially expressed genes in the HFD group. (**E**) GSEA enrichment analysis of the Cul3-RING ubiquitin ligase complex-related gene set. (**F**) GSEA enrichment analysis of the phosphatidylinositol 3-kinase complex-related gene set. *n* = 3 per group.

To explore the molecular mechanisms underlying HFD-induced skeletal muscle functional decline, transcriptome sequencing was performed on skeletal muscle from the NC and HFD groups. The volcano plot showed that 256 genes were upregulated and 127 genes were downregulated in the HFD group. Among these, inflammation- and immune-related genes, including *S100a8*, *Serpinc1*, *Serpina1c*, *Ngp* and *Camp*, were upregulated, whereas genes such as *Fasn*, *Cish* and *Kl* were downregulated (Figure 4A). GO analysis showed that the upregulated genes were enriched in terms related to immune–inflammatory responses, extracellular regions and binding functions, mainly involving innate immune response, inflammatory response, extracellular space and fatty acid binding (Figure 4B). KEGG analysis showed that the upregulated genes were significantly enriched in phagosome, cytokine–cytokine receptor interaction, complement and coagulation cascades, PPAR signalling pathway and lipid metabolism-related pathways (Figure 4C,D). GSEA further showed that the Cul3-RING ubiquitin ligase complex and phosphatidylinositol 3-kinase complex gene sets were negatively enriched in the HFD group (Figure 4E,F), suggesting that HFD may suppress skeletal muscle proteostasis and PI3K-related signalling. Together, these results indicate that HFD activates immune–inflammatory and lipid metabolism pathways in skeletal muscle while suppressing proteostasis and metabolic regulation, providing a molecular basis for skeletal muscle structural damage and exercise capacity decline.

### 3.4. Kukoamine B Downregulates Immune Inflammation-Related Transcriptional Features in Skeletal Muscle of HFD-Fed Mice

**Figure 5:**
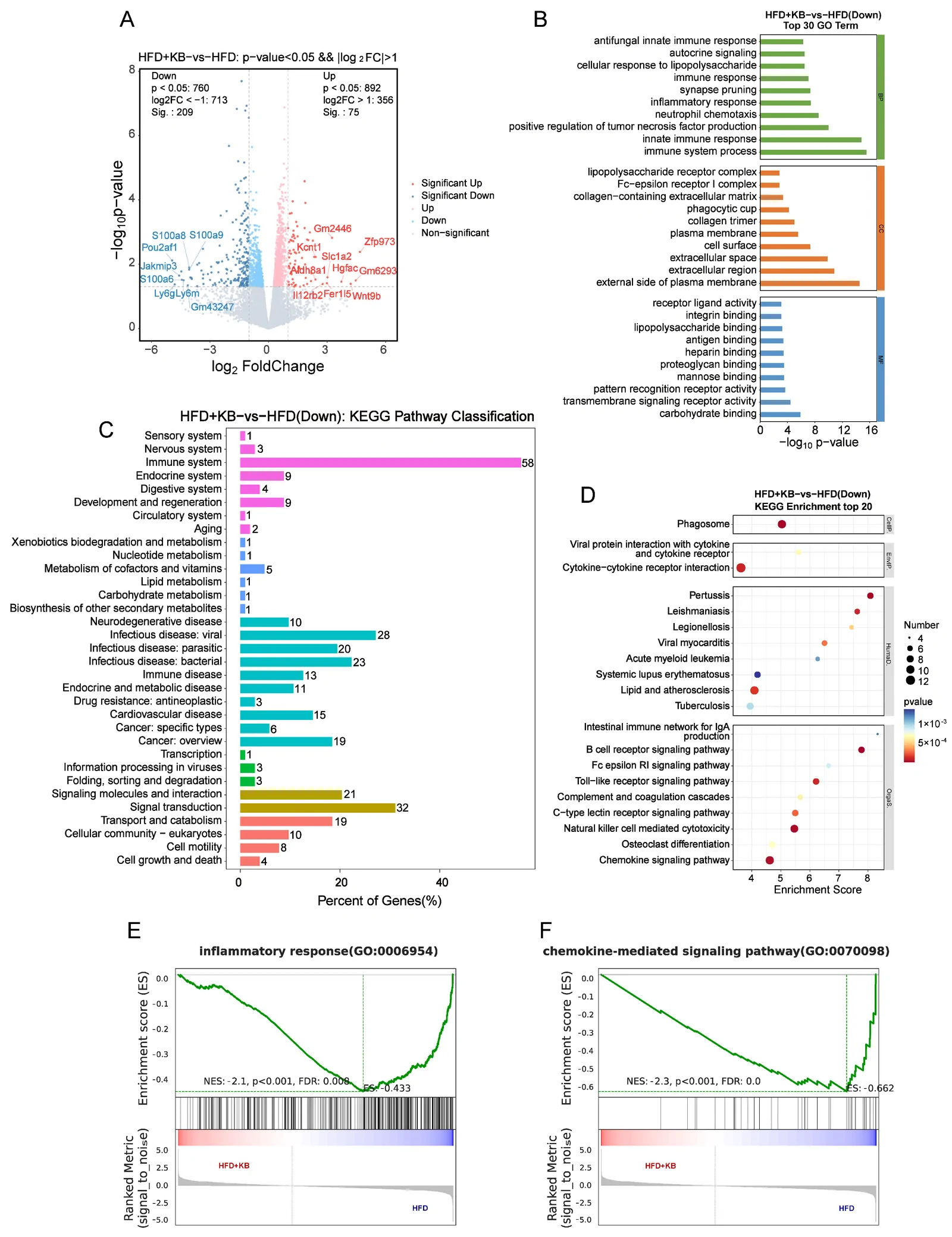
Kukoamine B downregulates immune inflammation-related transcriptional features in skeletal muscle of HFD-fed mice. (**A**) Volcano plot of differentially expressed genes in skeletal muscle between the HFD+KB and HFD groups. (**B**) GO functional enrichment analysis of significantly downregulated genes in the HFD+KB group compared with the HFD group. (**C**) KEGG pathway classification analysis of significantly downregulated genes in the HFD+KB group compared with the HFD group. (**D**) Bubble plot of KEGG enrichment analysis of significantly downregulated genes in the HFD+KB group compared with the HFD group. (**E**) GSEA enrichment analysis of the inflammatory response-related gene set. (**F**) GSEA enrichment analysis of the chemokine-mediated signalling pathway-related gene set. *n* = 3 per group.

To explore the molecular mechanisms by which KB improves skeletal muscle function in HFD-fed mice, transcriptome sequencing was further performed on skeletal muscle from the HFD+KB and HFD groups. The volcano plot showed that 75 genes were upregulated and 209 genes were downregulated in the HFD+KB group (Figure 5A). The downregulated genes included inflammation- and immune-related genes such as *S100a8*, *S100a9*, *Pou2af1*, *Jakmip3* and *S100a6*, suggesting that KB suppressed the HFD-induced inflammatory transcriptional response in skeletal muscle. GO analysis showed that the downregulated genes were mainly enriched in terms related to immune–inflammatory responses and lipopolysaccharide responses, including cellular response to lipopolysaccharide, immune response, inflammatory response and neutrophil chemotaxis (Figure 5B). KEGG analysis showed that the downregulated genes were enriched in pathways including phagosome, cytokine–cytokine receptor interaction, Toll-like receptor signalling, complement and coagulation cascades, chemokine signalling and B cell receptor signalling (Figure 5C,D), suggesting that KB suppressed immune–inflammatory, chemokine signalling and pattern recognition receptor-related pathways in skeletal muscle. GSEA further showed that the inflammatory response and chemokine-mediated signalling pathway gene sets were negatively enriched in the HFD+KB group (Figure 5E,F), supporting the conclusion that KB broadly downregulated inflammatory and chemokine signalling in skeletal muscle. Together, these results indicate that KB markedly suppresses HFD-induced immune inflammation-related transcriptional changes in skeletal muscle, especially genes and pathways related to LPS recognition, innate immunity, chemokines and inflammatory cytokine regulation.

### 3.5. Kukoamine B Reduces LPS Burden and Inhibits Activation of TLR4/NF-κB Inflammatory Signalling in Skeletal Muscle of HFD-Fed Mice

**Figure 6:**
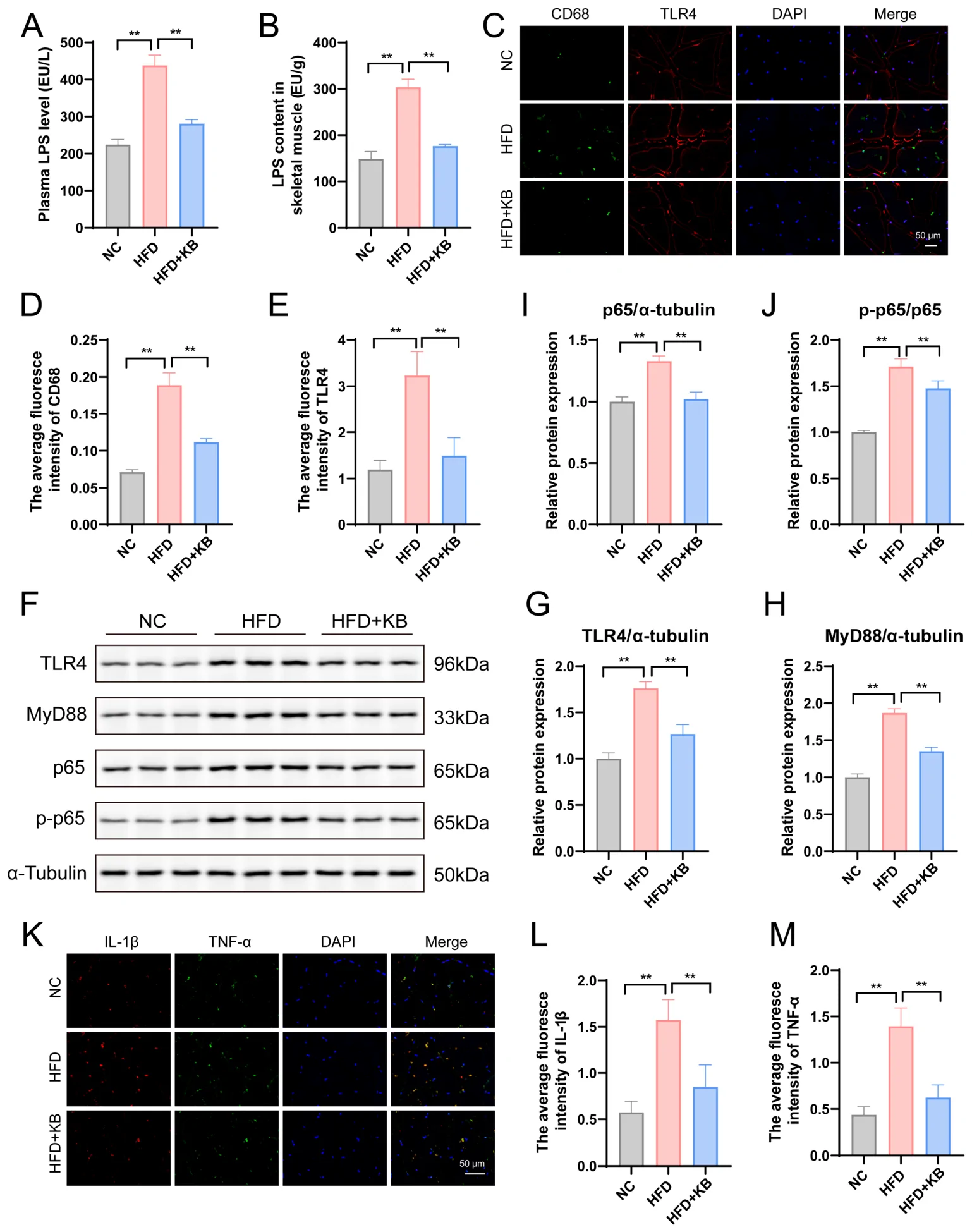
Kukoamine B inhibits HFD-induced activation of LPS/TLR4/NF-κB inflammatory signalling in skeletal muscle. (**A**) Plasma LPS levels. (**B**) Skeletal muscle LPS content. (**C**) Representative immunofluorescence images of CD68 and TLR4 in skeletal muscle. (**D**) Quantitative analysis of CD68 mean fluorescence intensity. (**E**) Quantitative analysis of TLR4 mean fluorescence intensity. (**F**) Representative Western blot images of TLR4, MyD88, p65, p-p65 and α-tubulin protein expression in skeletal muscle. (**G**) TLR4/α-tubulin protein expression. (**H**) MyD88/α-tubulin protein expression. (**I**) p65/α-tubulin protein expression. (**J**) p-p65/p65 ratio. (**K**) Representative immunofluorescence images of IL-1β and TNF-α in skeletal muscle. (**L**) Quantitative analysis of IL-1β mean fluorescence intensity. (**M**) Quantitative analysis of TNF-α mean fluorescence intensity. Data are presented as the mean ± SD. For panels (**A**,**B**), *n* = 6 per group; for panels (**D**,**E**,**L**,**M**), *n* = 4 per group; for panels (**G**–**J**), *n* = 5 per group. ** *p* < 0.01.

To verify whether KB improved skeletal muscle function by regulating LPS-related inflammatory signalling, plasma and skeletal muscle LPS levels were measured, and the TLR4/MyD88/NF-κB pathway and inflammatory cytokine expression in skeletal muscle were analyzed. The results showed that LPS levels in both plasma and skeletal muscle were significantly increased in the HFD group, whereas KB intervention markedly reduced both levels (Figure 6A,B). Immunofluorescence analysis showed enhanced CD68- and TLR4-positive signals in skeletal muscle of the HFD group. After KB intervention, the mean fluorescence intensities of CD68 and TLR4 were significantly decreased (Figure 6C–E), suggesting that KB attenuated the HFD-induced increases in CD68 immunoreactivity and TLR4 expression in skeletal muscle. Further Western blot analysis showed that HFD significantly upregulated TLR4 and MyD88 protein expression, increased total p65 abundance, and increased the p-p65/p65 ratio in skeletal muscle. After KB intervention, TLR4 and MyD88 expression, total p65 abundance and the p-p65/p65 ratio were markedly reduced (Figure 6F–J). Immunofluorescence analysis also showed that KB significantly reduced HFD-induced IL-1β- and TNF-α-positive signals in skeletal muscle (Figure 6K–M). Together, these findings indicate that KB reduces circulating and local skeletal muscle LPS burden in HFD-fed mice and attenuates TLR4/MyD88/NF-κB-related inflammatory signalling.

### 3.6. Kukoamine B Reduces LPS Burden and Attenuates TLR4/NF-κB-Related Inflammatory Signalling in Skeletal Muscle of HFD-Fed Mice

**Figure 7:**
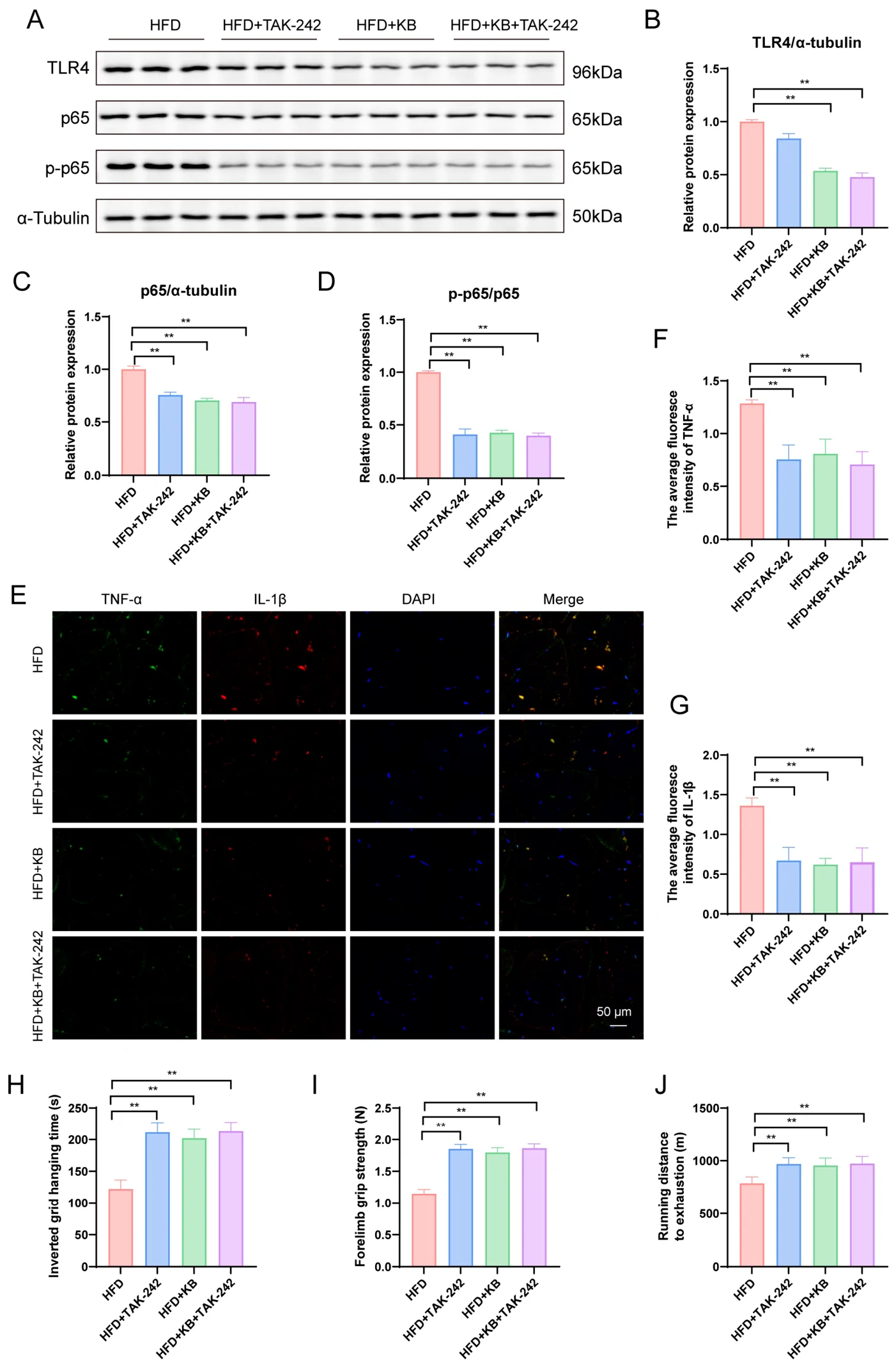
TAK-242 mimics the beneficial effects of kukoamine B on skeletal muscle inflammatory signalling and exercise capacity decline in HFD-fed mice. (**A**) Representative Western blot images of TLR4, p65, p-p65 and α-tubulin protein expression in skeletal muscle from each group. (**B**) TLR4/α-tubulin protein expression. (**C**) p65/α-tubulin protein expression. (**D**) p-p65/p65 ratio. (**E**) Representative immunofluorescence images of TNF-α and IL-1β in skeletal muscle. (**F**) Quantitative analysis of TNF-α mean fluorescence intensity. (**G**) Quantitative analysis of IL-1β mean fluorescence intensity. (**H**) Inverted grid hanging time. (**I**) Forelimb grip strength. (**J**) Running distance to exhaustion. Data are presented as the mean ± SD. For panels (**A**–**D**), *n* = 6 per group; for panels (**E**–**G**), *n* = 4 per group; for panels (**H**–**J**), *n* = 5 per group. ** *p* < 0.01.

To investigate the potential involvement of TLR4/NF-κB inflammatory signalling in the effects of KB on HFD-associated skeletal muscle dysfunction, the TLR4 inhibitor TAK-242 was used. Western blot analysis showed that TAK-242, KB and their combined treatment significantly reduced TLR4 and p65 protein expression and the p-p65/p65 ratio in skeletal muscle (Figure 7A–D). Immunofluorescence analysis further showed that these treatments reduced TNF-α and IL-1β signals in skeletal muscle (Figure 7E–G). Consistently, TAK-242 and KB improved inverted grid hanging time, forelimb grip strength and running distance to exhaustion, while the combination did not produce a statistically detectable additional effect under the conditions tested (Figure 7H–J). Two-way ANOVA further revealed significant main effects of KB and TAK-242 on most of the assessed inflammatory and exercise capacity-related outcomes, whereas no significant KB × TAK-242 interactions were detected (all interaction *p* > 0.05). Together, these findings indicate that TAK-242 produced effects similar to those observed with KB and support the potential involvement of TLR4/NF-κB inflammatory signalling in the beneficial effects associated with KB, without establishing this pathway as the primary mediator.

## 4. Discussion

This study systematically evaluated the effects of KB on skeletal muscle structure, inflammatory status and exercise capacity-related indicators in mice under HFD conditions. The results showed that KB not only reduced final body weight, epididymal fat weight and plasma TG and TC levels in HFD-fed mice but also improved inverted grid hanging time, forelimb grip strength and treadmill exhaustion performance. Further histological, transcriptomic and protein analyses showed that KB alleviated lipid deposition, collagen deposition and inflammatory responses in skeletal muscle and regulated AKT/mTOR-related protein metabolic signalling. In terms of mechanistic validation, KB reduced LPS levels in plasma and skeletal muscle, inhibited TLR4/NF-κB signalling, and decreased TNF-α and IL-1β expression. TAK-242 mimicked the beneficial effects of KB on skeletal muscle inflammatory signalling and exercise capacity-related indicators, while combined intervention with KB and TAK-242 did not produce a statistically detectable additional effect under the conditions tested. These findings suggest that the regulatory effect of KB on HFD-related skeletal muscle functional changes may be associated with suppression of LPS/TLR4/NF-κB inflammatory signalling.

The effect of HFD on exercise capacity is not merely a mechanical burden caused by body weight gain but may also result from changes in the local metabolic and inflammatory microenvironment of skeletal muscle. Previous studies have shown that HFD impairs skeletal muscle performance, manifesting as reduced muscle strength, increased fatigue or decreased endurance [32,33]. In the present study, the absolute quadriceps weight did not change markedly in the HFD group, whereas the quadriceps weight/body weight ratio decreased. At the same time, inverted grid hanging time, grip strength and treadmill exhaustion performance were all reduced. These findings suggest that skeletal muscle dysfunction under HFD conditions does not necessarily depend on an obvious reduction in muscle weight but may be associated with relative insufficiency of muscle mass, lipid deposition, tissue structural abnormalities and inflammatory activation. Similar to the findings of Yoo et al. on HFD-induced impairment of skeletal muscle performance [34], the present study further showed that KB improved HFD-related exercise capacity decline.

It should be noted that KB also reduced body weight and adiposity in HFD-fed mice; therefore, the improvements in exercise capacity and skeletal muscle phenotypes may partly reflect improvements in systemic metabolic status. In particular, inverted grid performance can be influenced by body weight loading, whereas treadmill exhaustion reflects integrated whole-body exercise capacity. Moreover, food and energy intake were not systematically recorded, and an HFD pair-fed or weight-matched control group was not included. Thus, the present findings do not establish a direct effect of KB on skeletal muscle independent of changes in body weight and systemic metabolism. Future studies incorporating pair-fed or weight-matched controls and direct assessments of muscle contractile function are warranted to clarify this issue. Structural changes in skeletal muscle provide an important basis for impaired muscle strength and exercise endurance. In this study, HFD increased the Oil Red O-positive area and Sirius Red-positive collagen area in skeletal muscle, whereas KB intervention markedly reduced both lipid deposition and collagen deposition. Ectopic lipid deposition in skeletal muscle can affect fatty acid oxidation, insulin signalling and mitochondrial function and is associated with reduced muscle contractile efficiency [35,36]. In addition, extracellular matrix remodelling and collagen deposition can alter the mechanical properties of muscle tissue and affect force transmission, myofibre repair and regeneration [37]. Therefore, the improvement of lipid deposition and collagen deposition by KB may provide a histological basis for its effects on grip strength and exercise endurance.

This study found that HFD reduced AKT, p-AKT/AKT, mTOR and p-mTOR/mTOR levels in skeletal muscle, whereas KB intervention partially restored AKT/mTOR-related signalling. The AKT/mTOR pathway is an important signalling axis that regulates skeletal muscle protein synthesis, cell growth and metabolic adaptation, and reduced activity of this pathway is generally associated with impaired muscle repair capacity and functional decline [38]. Notably, FBXO32 and MuRF-1 protein expression decreased in the HFD group and increased after KB intervention in the present study, which is not fully consistent with the increase in FBXO32/MuRF-1 commonly observed in classical rapid muscle atrophy models [39]. This difference suggests that the HFD condition in this study may not represent a typical muscle atrophy process characterized by enhanced protein degradation but may instead reflect an imbalance between protein synthesis and degradation. KB-induced upregulation of FBXO32 and MuRF-1 may help skeletal muscle protein synthesis and degradation reach a new balance. Zhao et al. [40] found that both skeletal muscle protein synthesis and degradation decreased in ageing mice, whereas exercise intervention increased both protein synthesis and degradation and improved exercise capacity. This phenomenon is similar to the findings of the present study, suggesting that these interventions may allow protein synthesis and degradation to reach a new and healthier balance. However, the mechanism by which KB regulates the balance between skeletal muscle protein synthesis and degradation requires further investigation.

The transcriptomic results further showed that HFD induced a clear immune–inflammatory activation profile in skeletal muscle. In this study, genes upregulated in the HFD group were mainly enriched in pathways and terms including innate immune response, inflammatory response, phagosome, cytokine–cytokine receptor interaction and complement and coagulation cascades. After KB intervention, the downregulated genes were mainly enriched in inflammatory response, the chemokine-mediated signalling pathway, cellular response to lipopolysaccharide, and the Toll-like receptor signalling pathway. These findings indicate that KB did not merely regulate a single inflammatory protein but broadly downregulated the immune–inflammatory programme in skeletal muscle at the transcriptional level. Previous studies have reported that skeletal muscle inflammation is an important component of obesity- and HFD-related insulin resistance and metabolic dysfunction [41], and that interventions such as exercise can improve the metabolic status of HFD-fed mice by suppressing chronic inflammation in skeletal muscle [42]. The present study is consistent with these findings and further suggests that KB, as a natural bioactive compound, may improve exercise capacity-related phenotypes by reshaping the skeletal muscle immune microenvironment.

Among the differentially expressed genes, changes in *S100a8* and *S100a9* are noteworthy. In this study, KB intervention markedly downregulated inflammation-related genes such as *S100a8* and *S100a9*. S100A8/S100A9 belongs to damage-associated molecular patterns, can amplify inflammatory responses, and is associated with activation of TLR4 and the NLRP3 inflammasome [43]. Franz et al. [44] showed that S100A9 participates in obesity-related macrophage dysfunction and can promote IL-1β release through TLR4–NLRP3-related mechanisms, impairing macrophage development and inducing the pro-inflammatory function of these cells. Therefore, KB-induced downregulation of *S100a8/S100a9* may not only reflect reduced inflammation but may also suggest inhibition of a local damage-associated inflammatory amplification loop in skeletal muscle. However, this study did not assess S100A8/S100A9 protein expression or verify their direct relationship with TLR4 signalling. Future studies may consider S100A8/S100A9 as candidate targets to further investigate their roles in KB-mediated regulation of skeletal muscle inflammation. Although selected DEGs were not independently validated by RT-qPCR, the inflammation-related transcriptomic changes were consistent with subsequent protein-level findings involving TLR4/MyD88/NF-κB signalling and downstream inflammatory mediators. Nevertheless, the lack of gene-level RT-qPCR validation represents a limitation of the present study.

LPS/TLR4/NF-κB signalling is a key component of the mechanistic axis examined in this study. HFD can increase circulating LPS levels by affecting the gut microbiota and intestinal barrier function, thereby inducing metabolic endotoxemia and low-grade inflammation in multiple tissues [45,46,47]. After recognition by TLR4, LPS can activate NF-κB through the MyD88-dependent pathway and promote the expression of pro-inflammatory cytokines such as TNF-α and IL-1β [48,49]. In the present study, HFD increased LPS levels in both plasma and skeletal muscle and activated TLR4/NF-κB signalling in skeletal muscle. After KB intervention, LPS burden, TLR4/NF-κB signalling and inflammatory cytokine expression were all markedly reduced. Previous studies have shown that KB can bind to and neutralize LPS and reduce LPS-induced inflammatory responses [24]. In LPS-induced inflammatory models, KB can also reduce plasma LPS levels and inhibit inflammatory cytokine expression [25]. Compared with these studies, the distinction of the present study lies in the fact that previous research mainly focused on sepsis or systemic metabolic abnormalities, whereas this study extended the LPS-related anti-inflammatory effect of KB to skeletal muscle functional changes under HFD conditions. By simultaneously measuring plasma LPS, skeletal muscle LPS and skeletal muscle TLR4/NF-κB signalling, this study provides more direct histological and molecular evidence for the effect of KB on HFD-related exercise capacity decline.

The TAK-242 experiment further strengthened the evidence that TLR4/NF-κB signalling is involved in the action of KB. TAK-242 is a selective inhibitor of TLR4 signalling that binds to TLR4 and interferes with its interaction with downstream adaptor proteins, thereby blocking TLR4-mediated inflammatory signal transduction [50]. In this study, both TAK-242 and KB reduced skeletal muscle TLR4/NF-κB signalling and TNF-α and IL-1β expression and improved inverted grid hanging time, forelimb grip strength and treadmill running distance to exhaustion. More importantly, combined intervention with KB and TAK-242 did not produce a statistically detectable additional effect under the conditions tested compared with KB intervention alone. This similar effect supports the potential involvement of TLR4/NF-κB inflammatory signalling in the action of KB. Ono et al. [51] also showed that TAK-242 attenuated endotoxemia-induced skeletal muscle atrophy, suggesting that blockade of TLR4 signalling has protective significance in inflammation-related skeletal muscle injury. However, these findings do not establish TLR4/NF-κB signalling as the sole or primary mediator of the protective effects of KB. Pharmacological inhibition by TAK-242 is not equivalent to genetic knockout, and other mechanisms, including the antioxidant effects of KB, regulation of lipid metabolism and protection of the intestinal barrier, may also contribute to its beneficial effects on skeletal muscle. Therefore, the present findings support TLR4/NF-κB signalling as a contributing mechanism associated with the effects of KB. Whether and to what extent the protective effects of KB depend on this pathway require further investigation using genetic approaches.

Several additional limitations should also be acknowledged. Formal investigator blinding was not implemented during histological, immunofluorescence and Western blot analyses, which should be considered when interpreting these findings. In addition, although purified KB was used in the present study, KB is a naturally occurring polyamine alkaloid derived from Lycium species. Therefore, these findings provide mechanistic evidence for the further development of Lycium-derived phytochemicals as potential pharmacological candidates for HFD-associated skeletal muscle dysfunction. Future studies are needed to evaluate the pharmacokinetic profile, bioavailability, dose–response relationship, long-term safety and efficacy of KB in standardized Lycium-derived preparations.

## 5. Conclusions

In conclusion, this study demonstrates that KB alleviates dyslipidemia, fat deposition, skeletal muscle structural damage and functional decline in HFD-fed mice while suppressing skeletal muscle immune–inflammatory responses at both the transcriptional and protein levels. These effects were associated with reduced LPS burden, inhibition of TLR4/MyD88/NF-κB signalling and decreased TNF-α and IL-1β expression. Pharmacological inhibition with TAK-242 further suggests that the TLR4/NF-κB pathway may be involved in the protective effects of KB against HFD-associated skeletal muscle dysfunction. These findings extend the current understanding of the pharmacological actions of KB in diet-induced metabolic injury and support KB as a promising Lycium-derived phytochemical for the management of metabolic inflammation-associated skeletal muscle dysfunction.

## Data Availability

The data supporting the findings of this study are available within the article and its Supplementary Information. Additional data are available from the corresponding author upon reasonable request.

## Supplementary Materials

The following supporting information can be downloaded at: https://www.mdpi.com/article/10.3390/biom16091275/s1. Supplementary Tables: The complete differential expression; Supplementary Figures: Original Western blot images.
