# Supplementary material for: Kukoamine B, a Lycium-Derived Polyamine Alkaloid, Attenuates High-Fat-Diet-Induced Skeletal Muscle Dysfunction with the Involvement of LPS/TLR4/NF-κB-Mediated Metabolic Inflammation
Source: Biomolecules. 2026 Sep 3;16(9):1275. doi: 10.3390/biom16091275 (PMC13604861; doi:10.3390/biom16091275)

mTOR Bright-field image

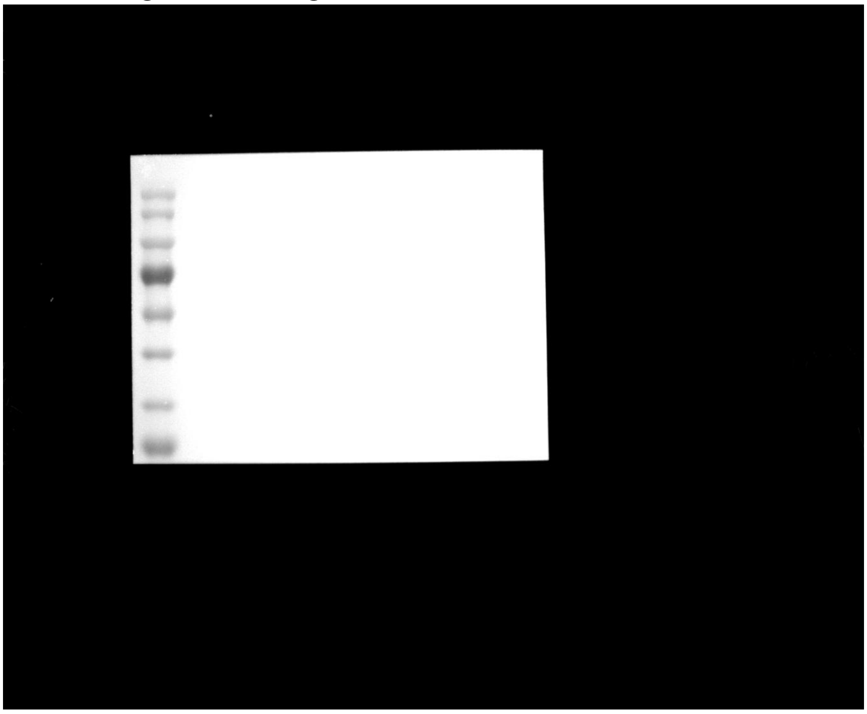

mTOR Chemiluminescence image

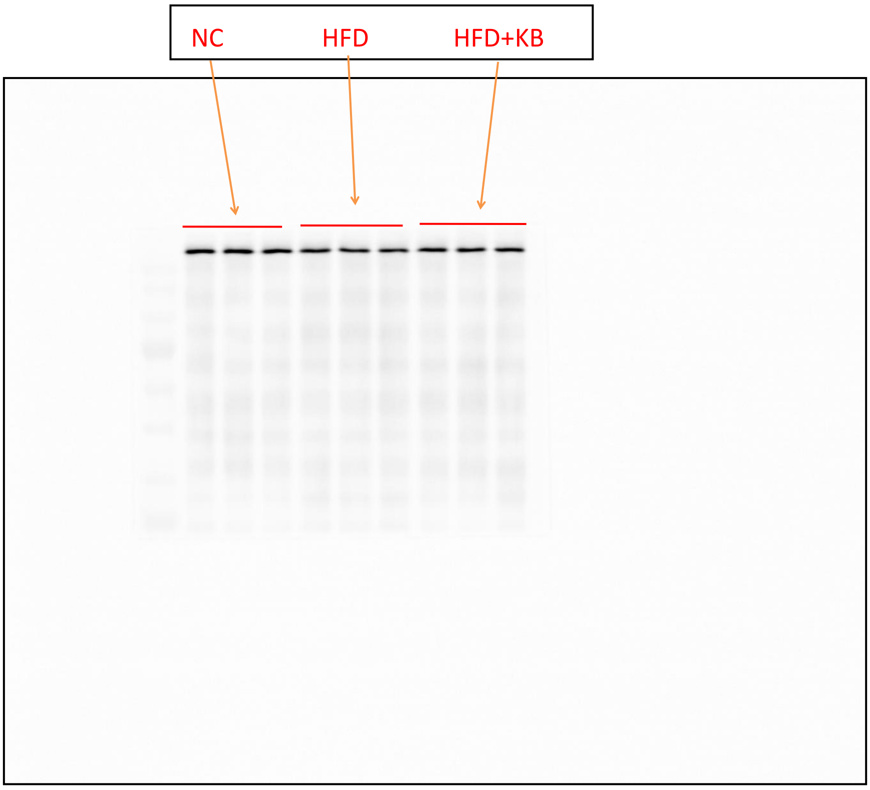

mTOR Merged image

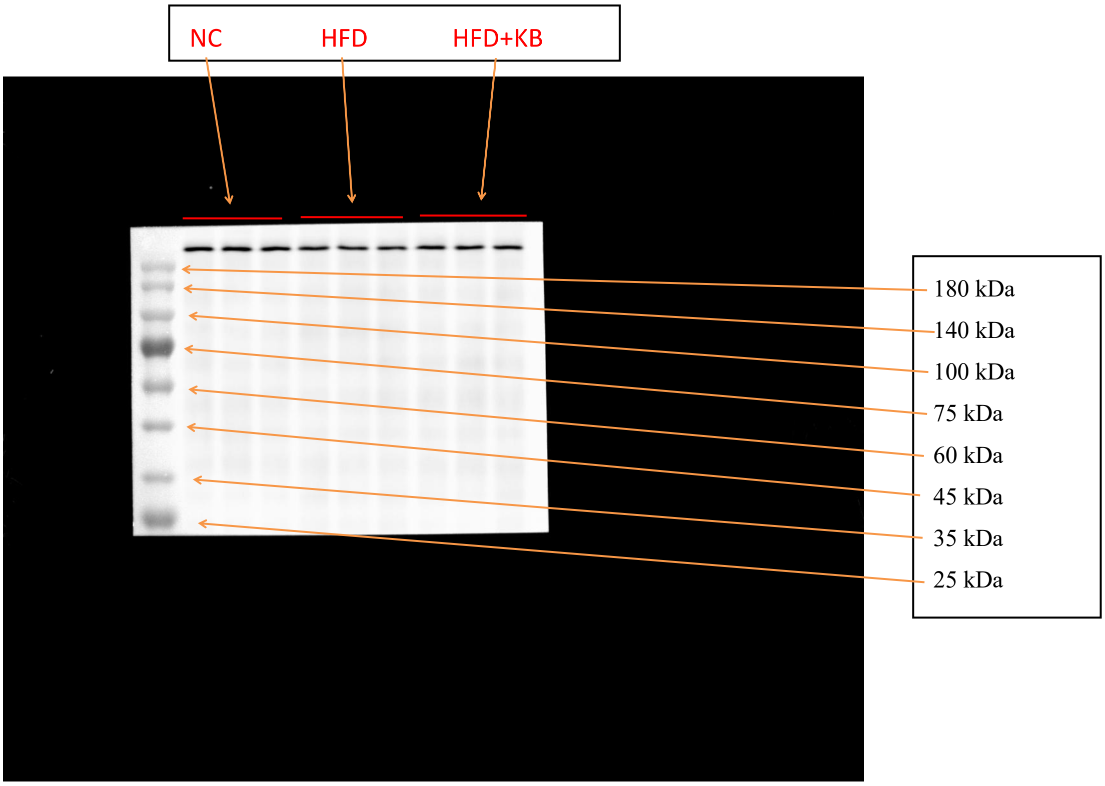

P-mTOR Bright-field image

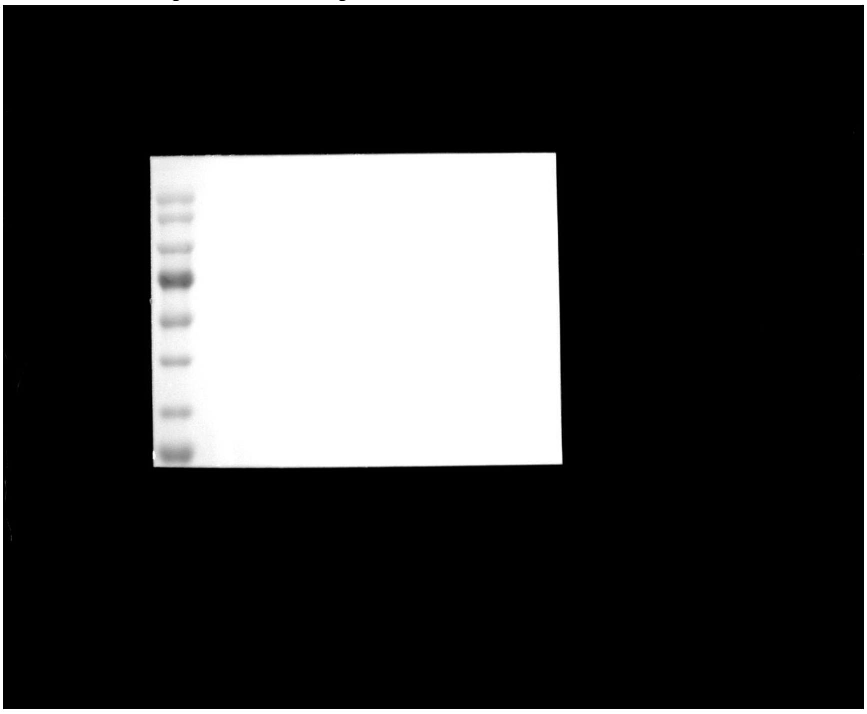

p-mTOR Chemiluminescence image

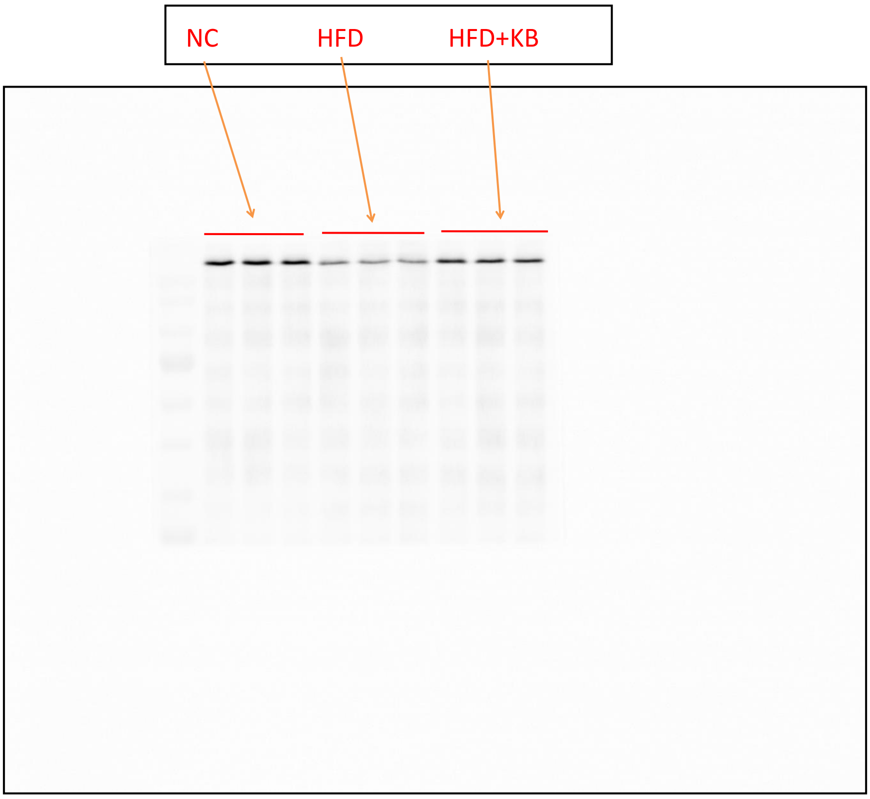

p-mTOR Merged image

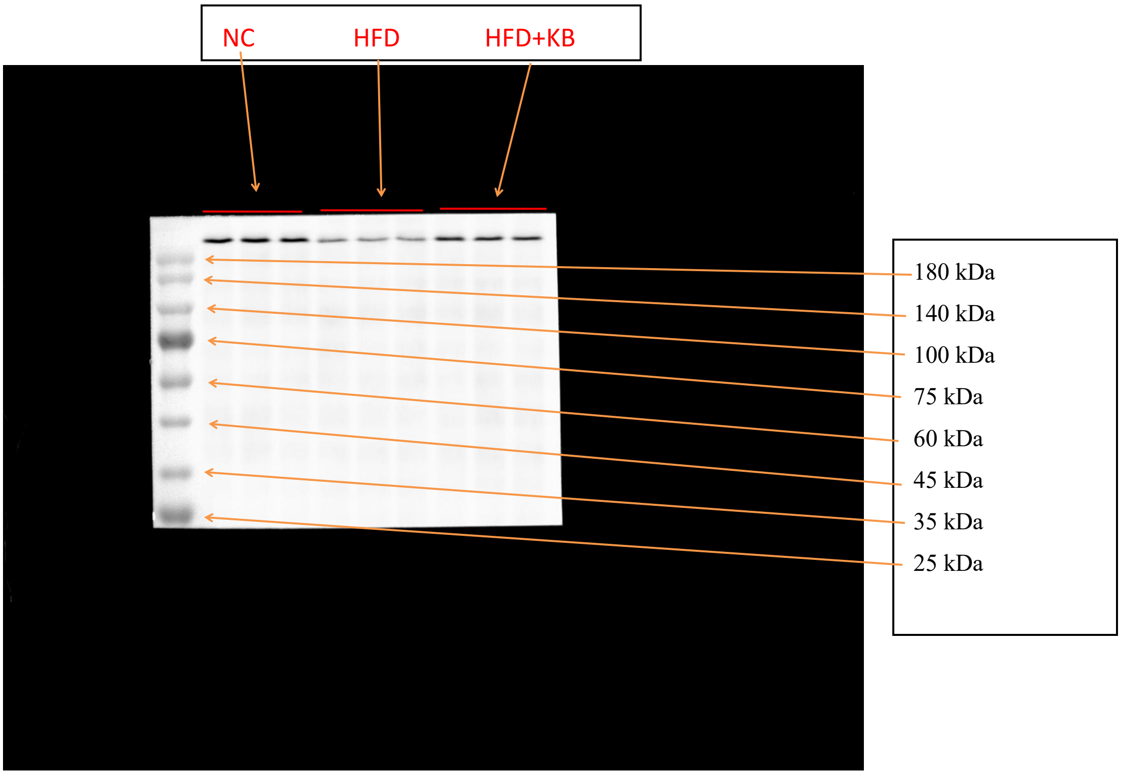

AKT Bright-field image

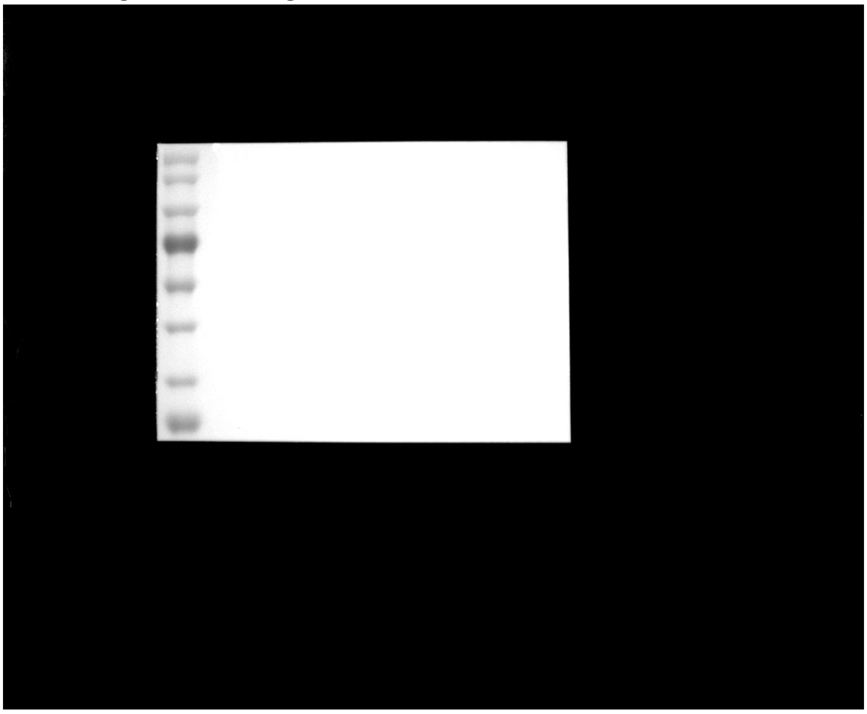

AKT Chemiluminescence image

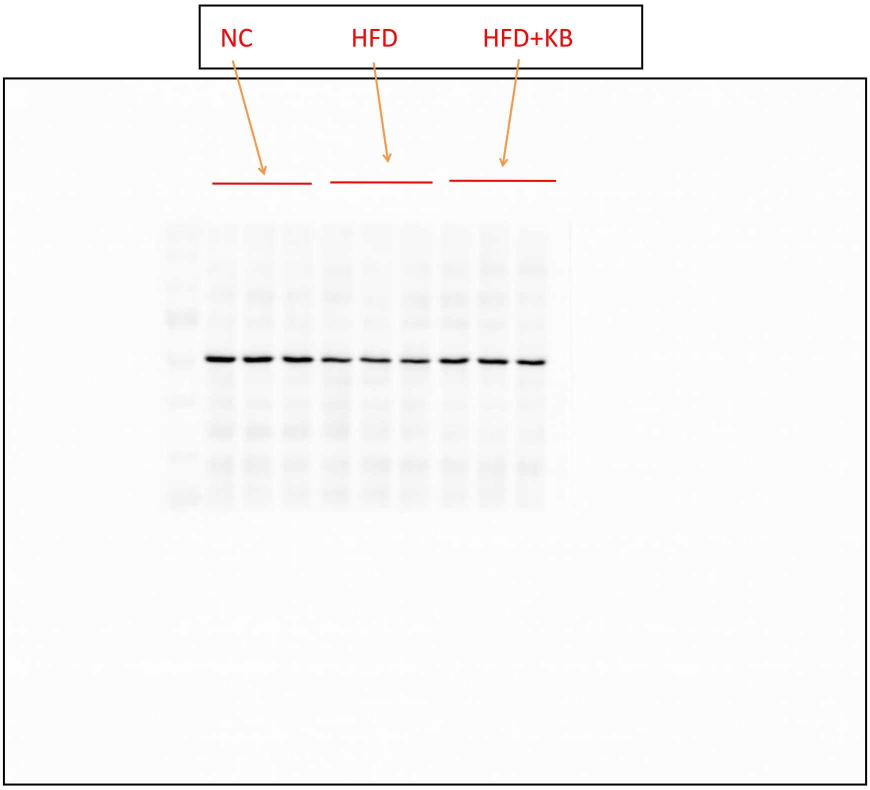

AKT Merged image

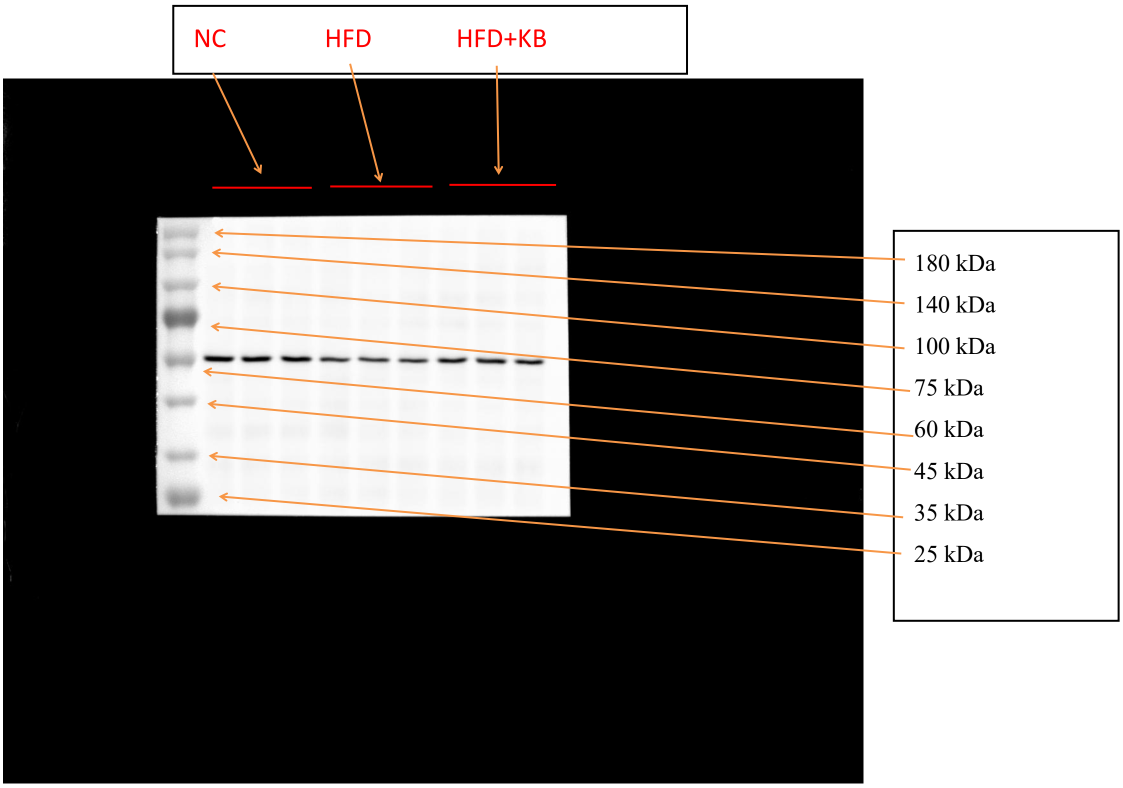

p-AKT Bright-field image

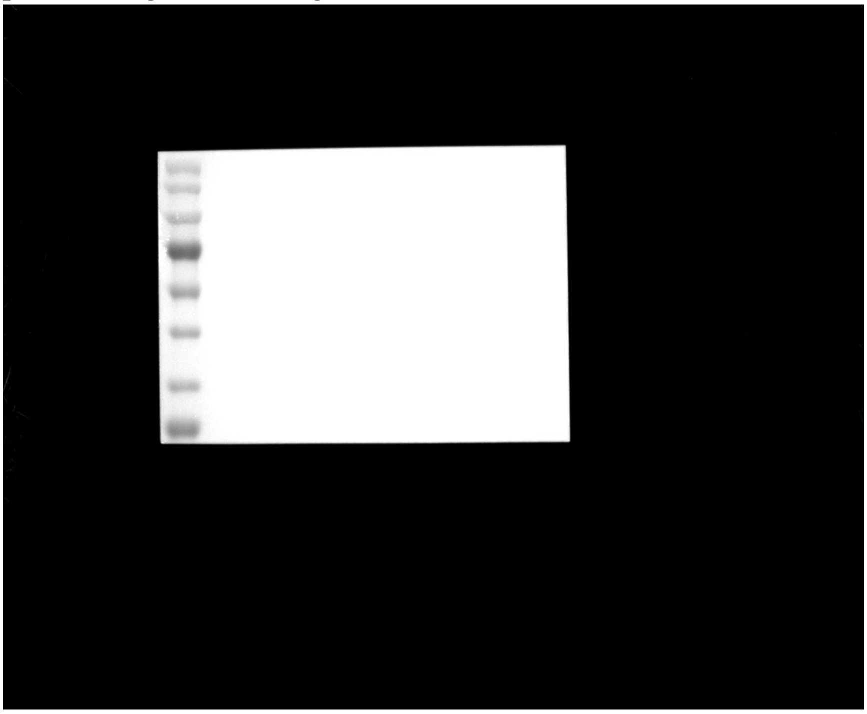

p-AKT Chemiluminescence image

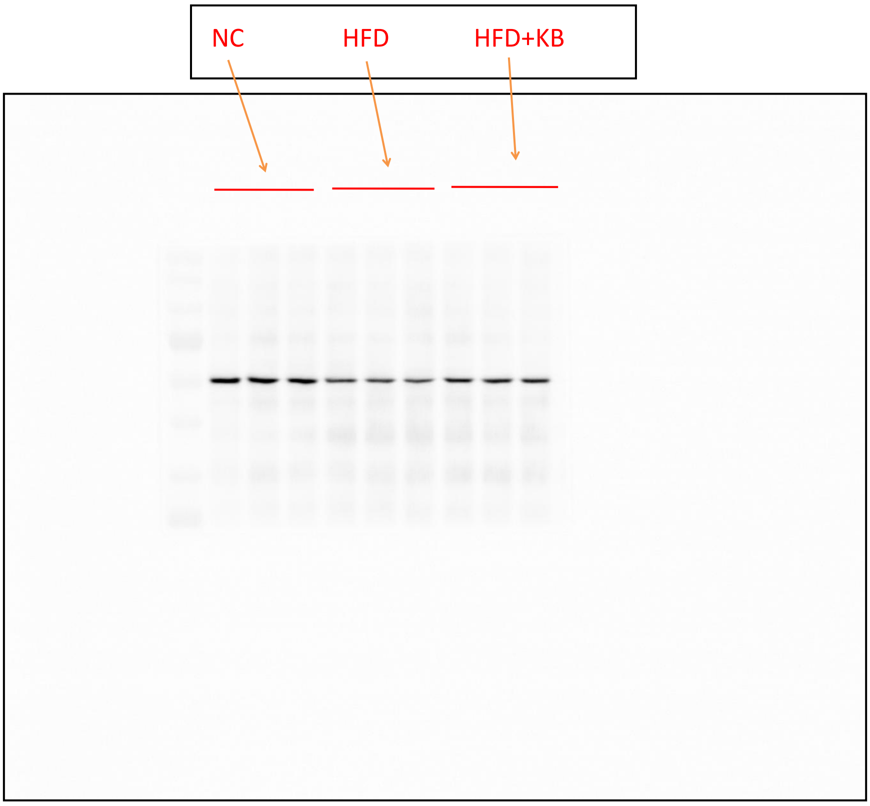

p-AKT Merged image

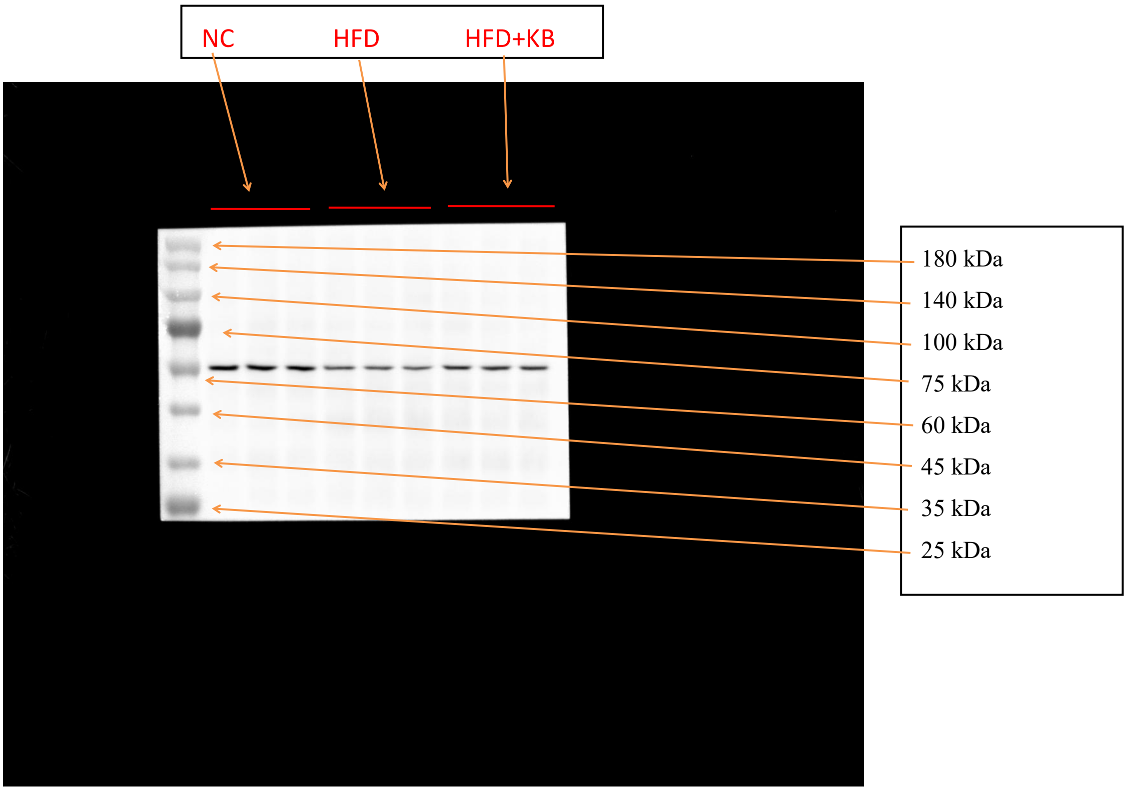

FBXO32 Bright-field image

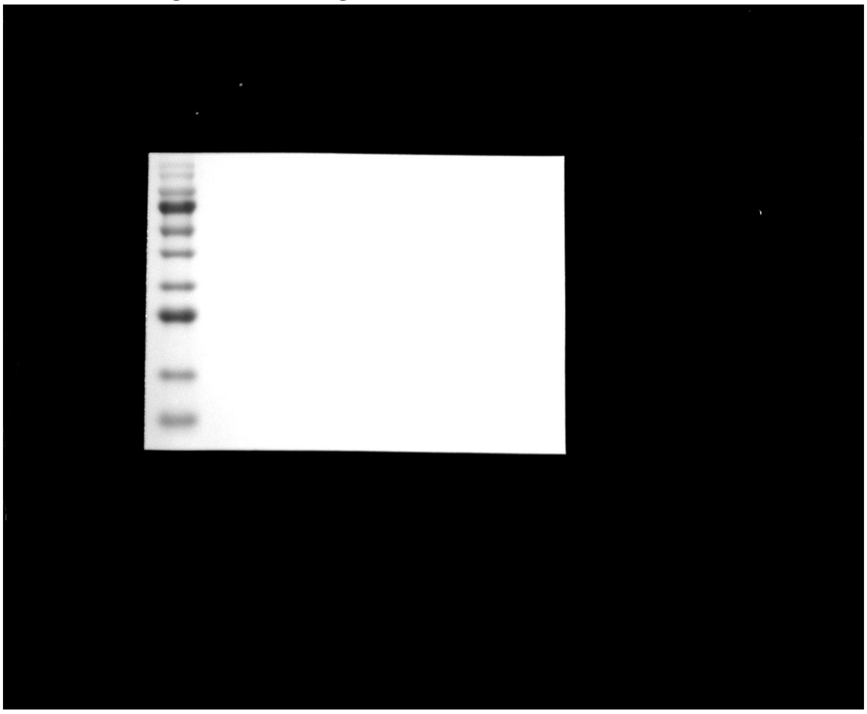

FBXO32 Chemiluminescence image

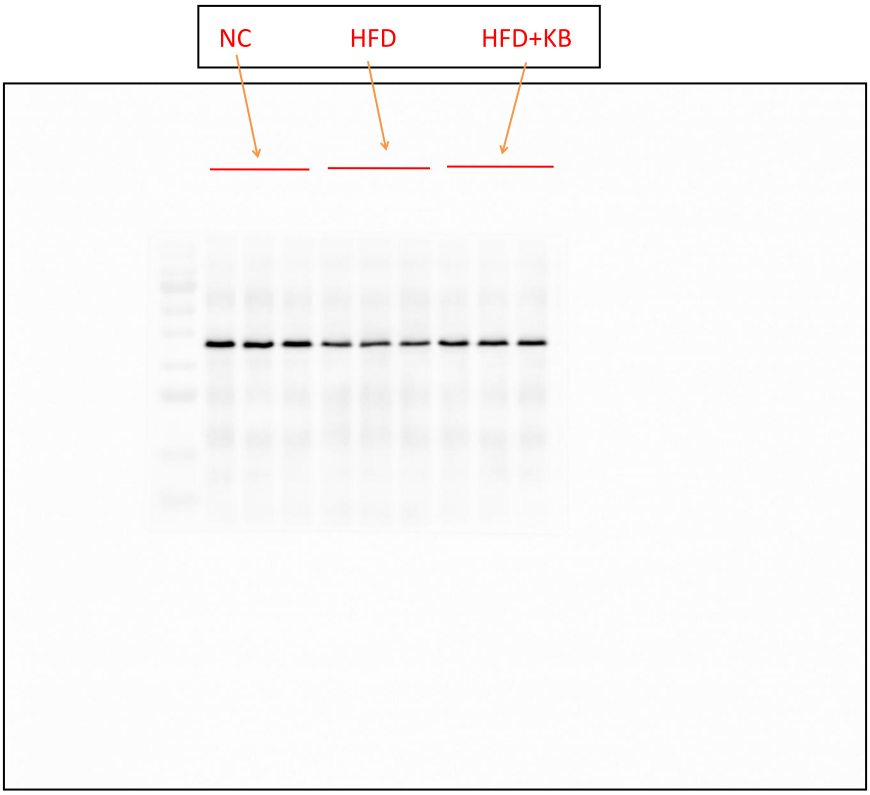

FBXO32 Merged image

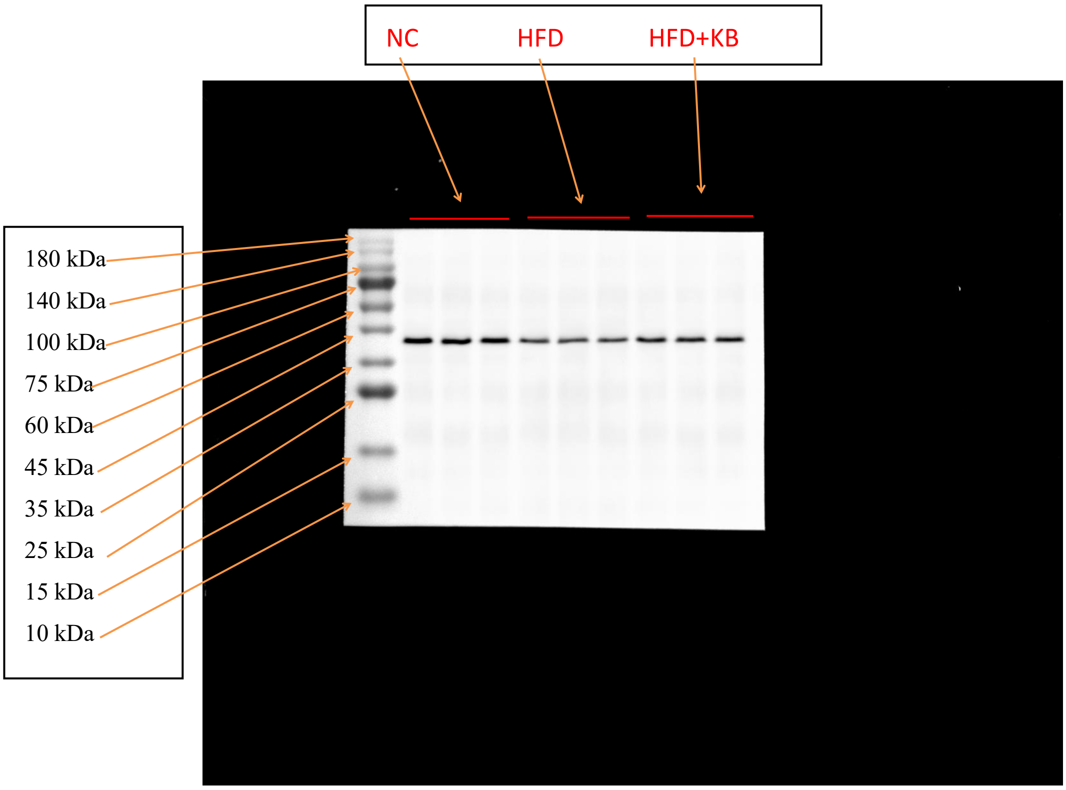

MuRF-1 Bright-field image

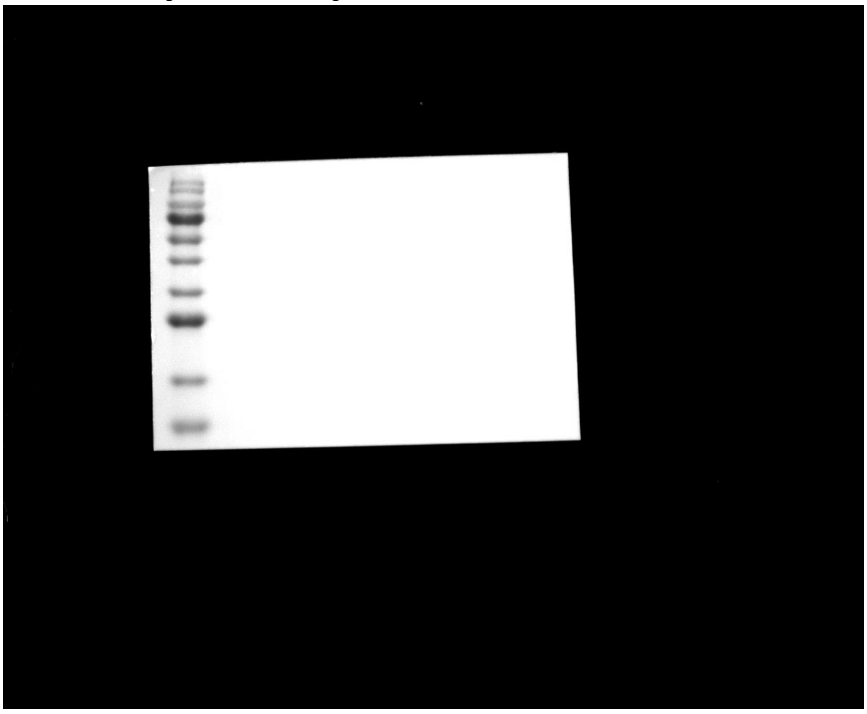

MuRF-1 Chemiluminescence image

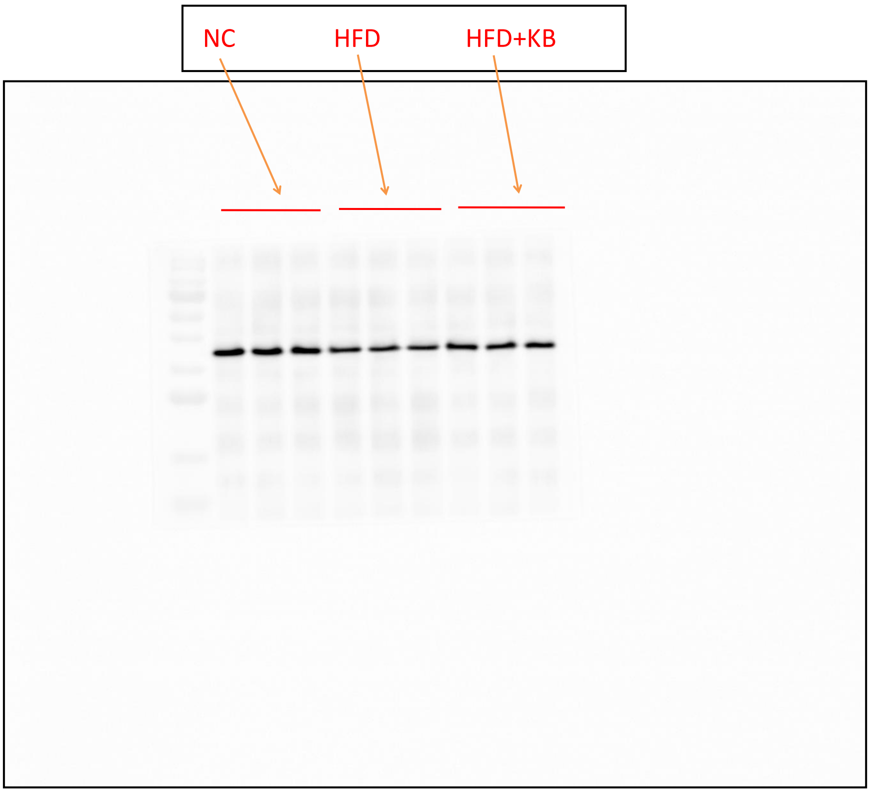

MuRF-1 (40kDa) Merged image

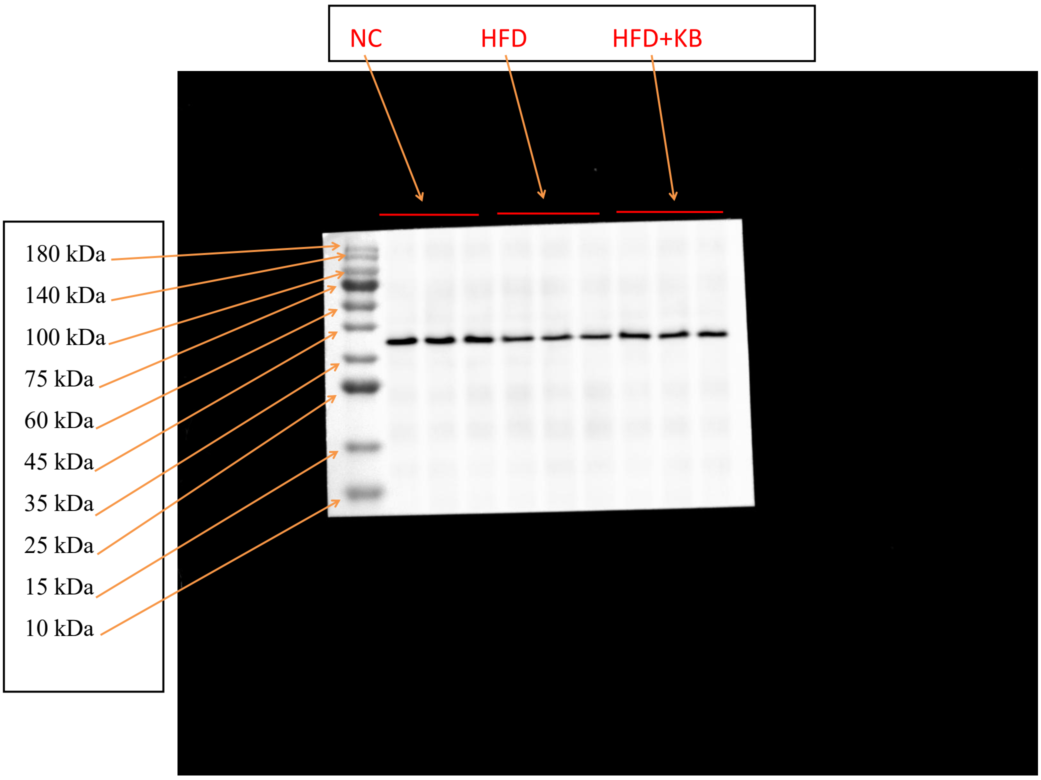

α-tubulin Bright-field image

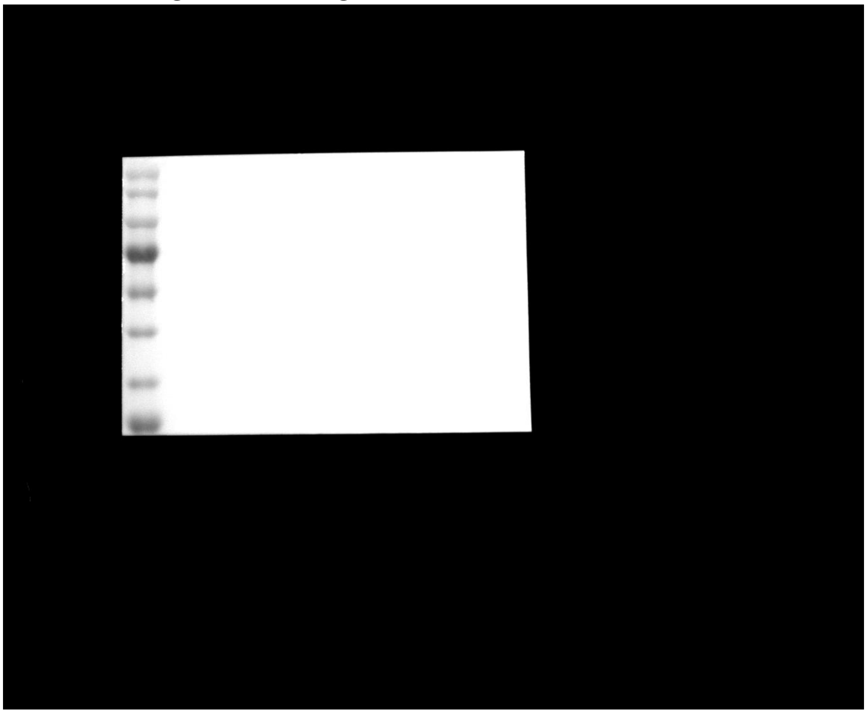

α-tubulin Chemiluminescence image

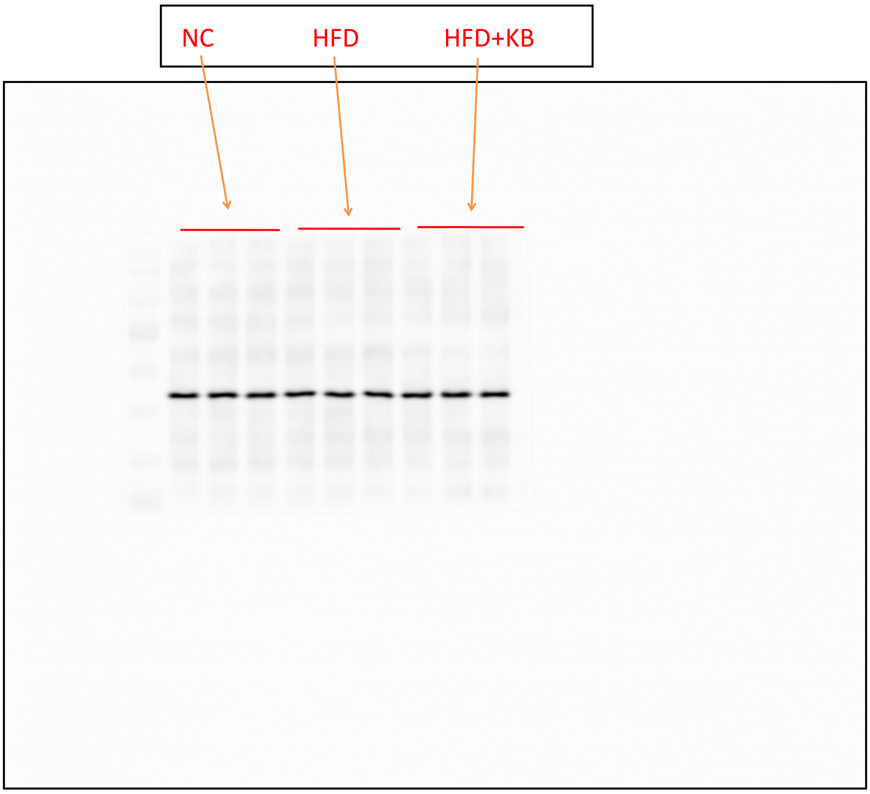

α-tubulin (50kDa) Merged image

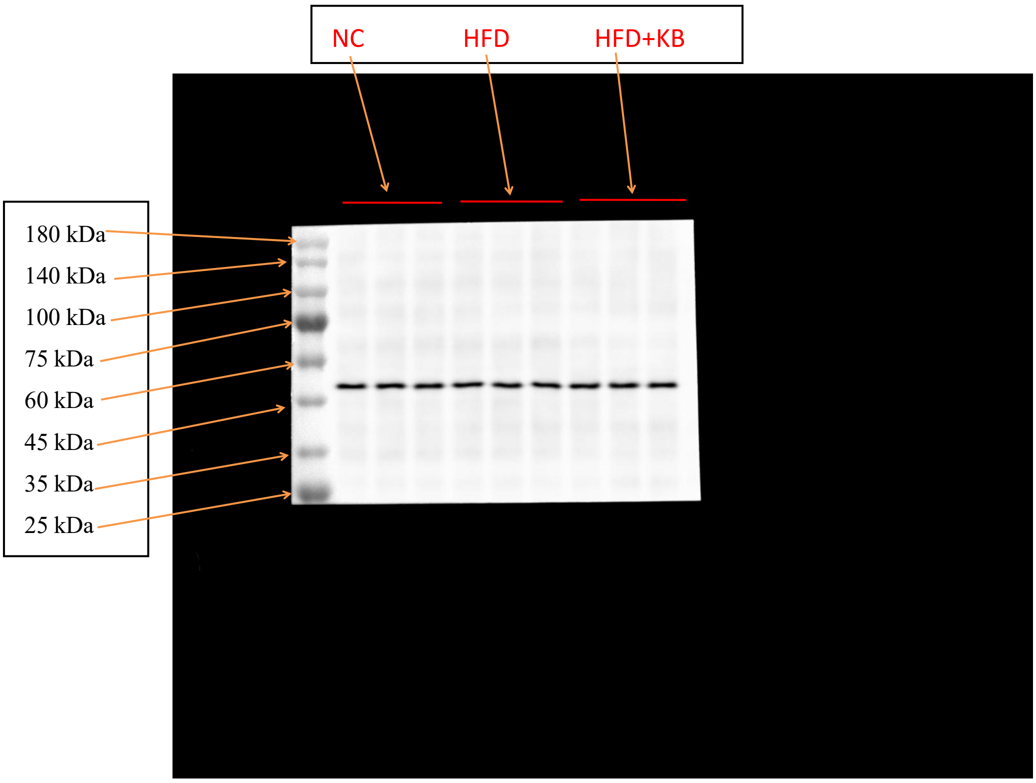

TLR4 Bright-field image

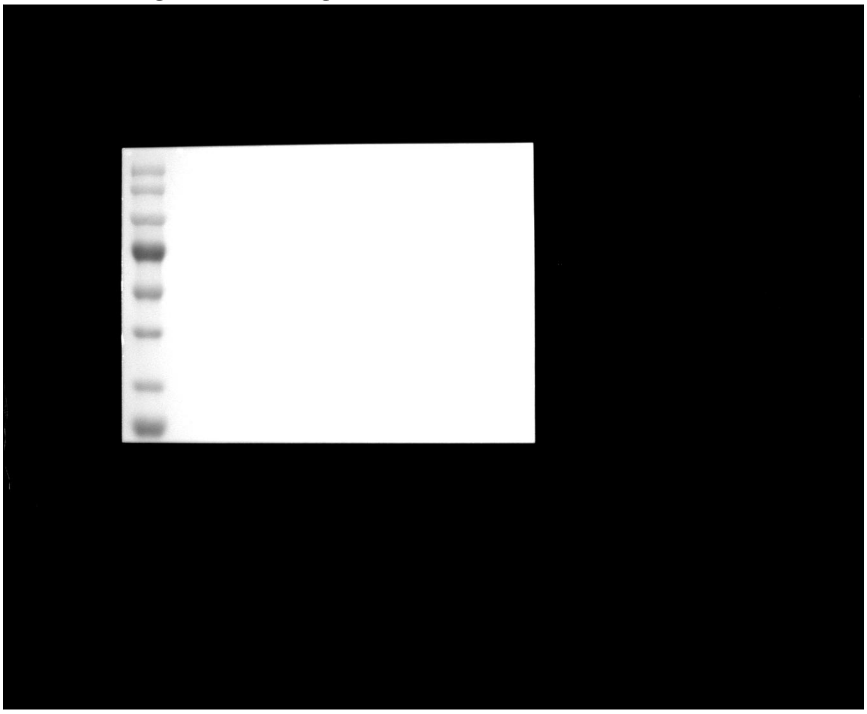

TLR4　Chemiluminescence image

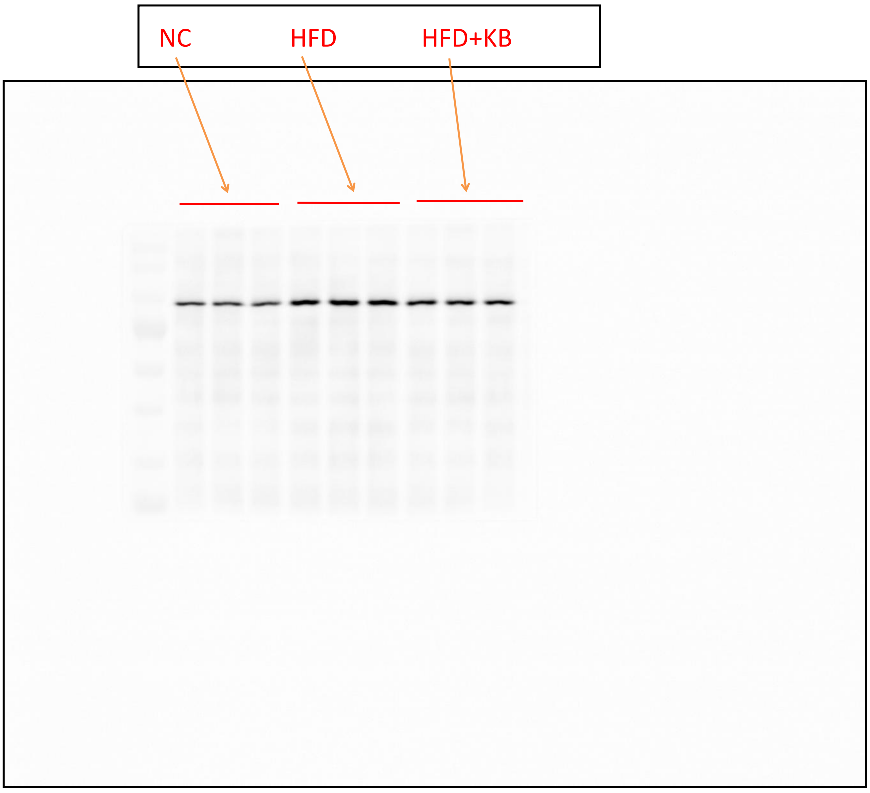

TLR4　(96kDa) Merged image

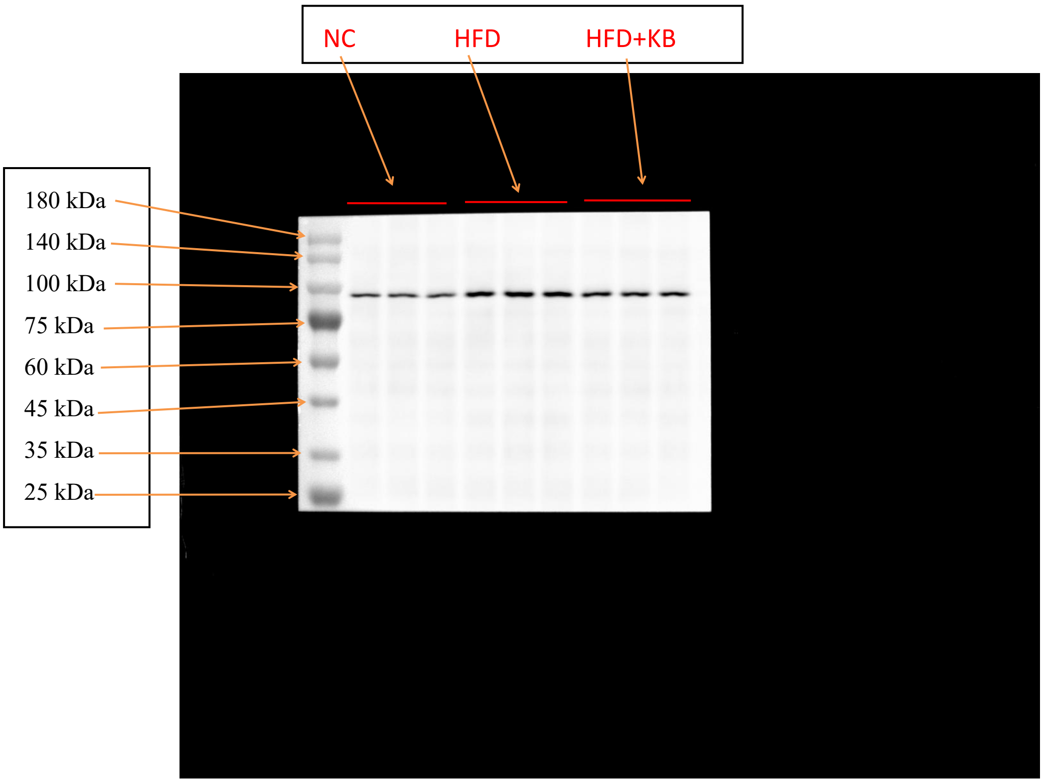

MyD88　Bright-field image

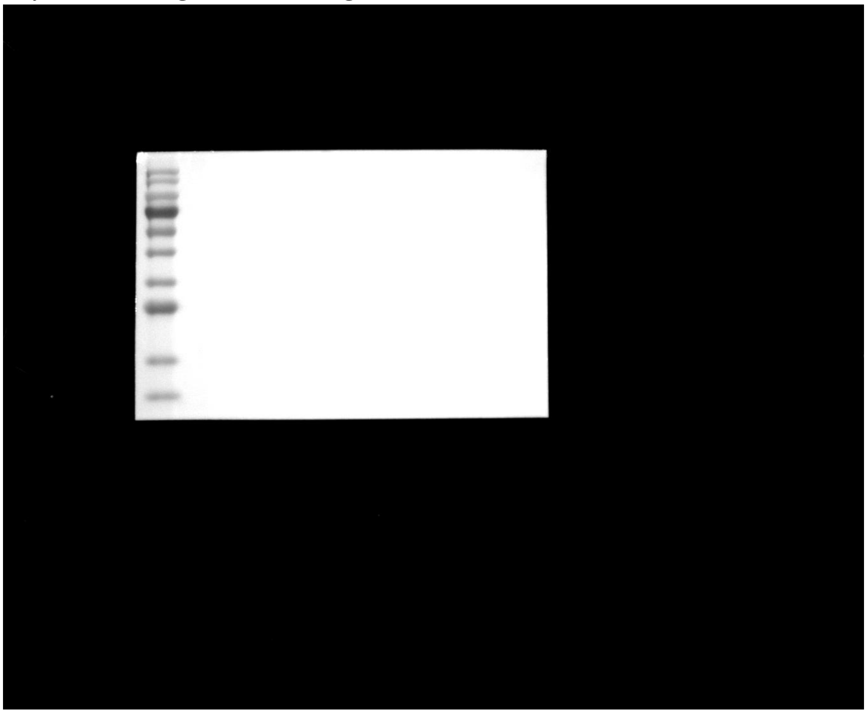

MyD88　Chemiluminescence image

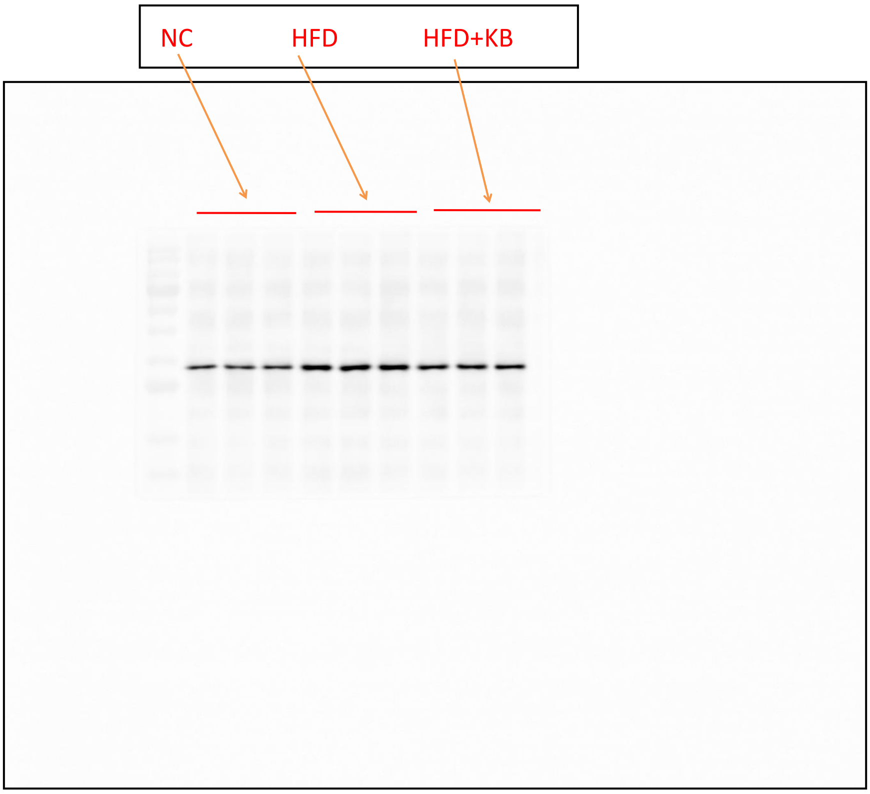

MyD88 (33kDa) Merged image

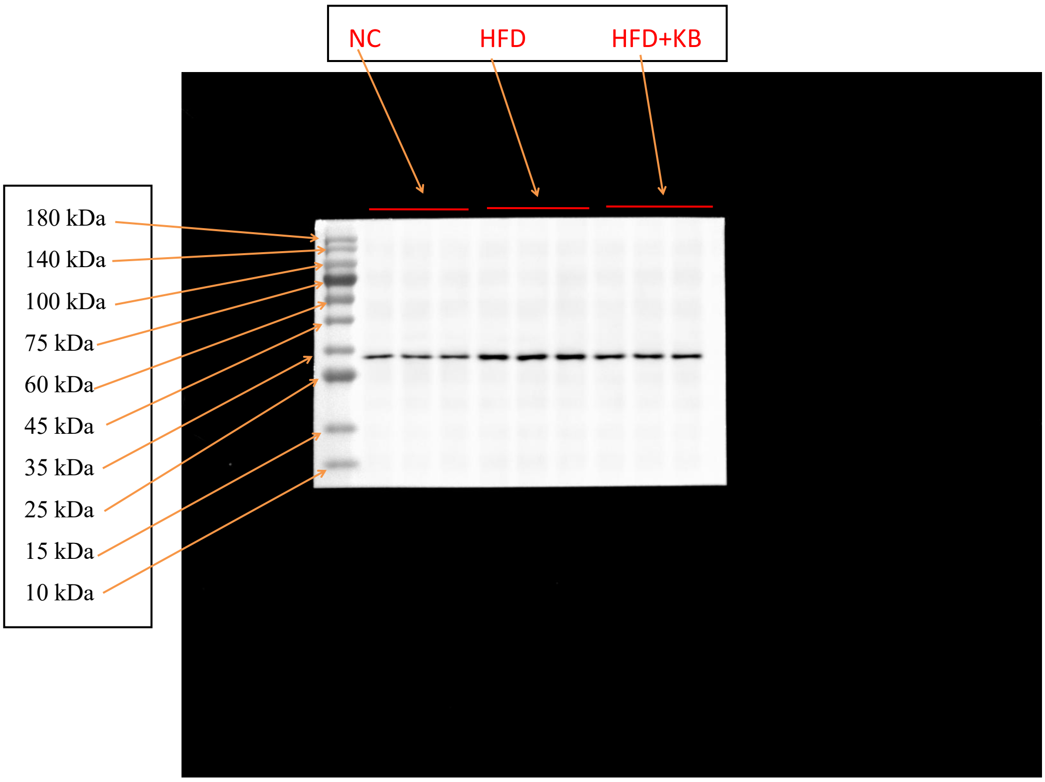

p65 Bright-field image

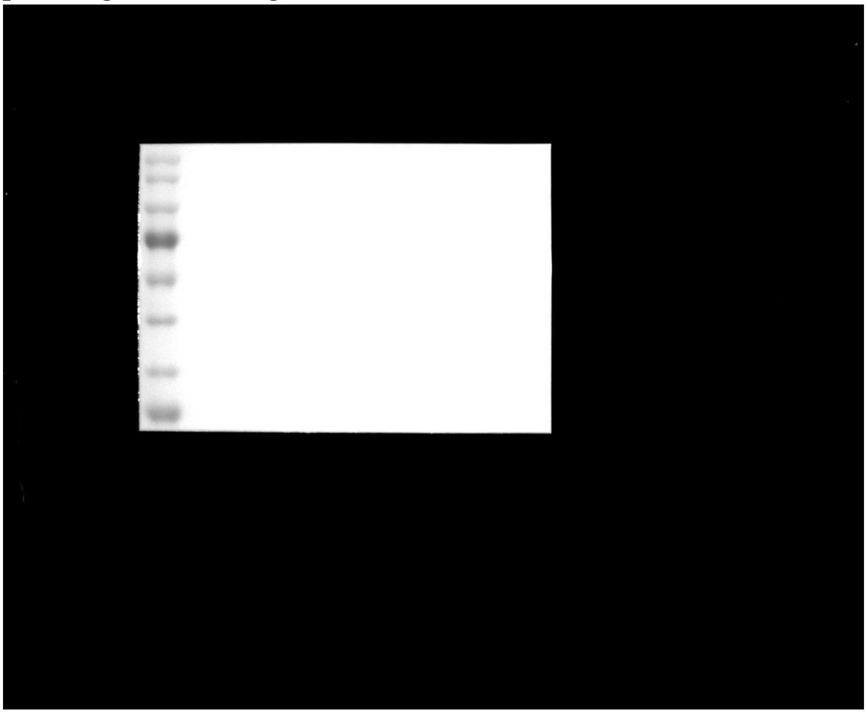

p65 Chemiluminescence image

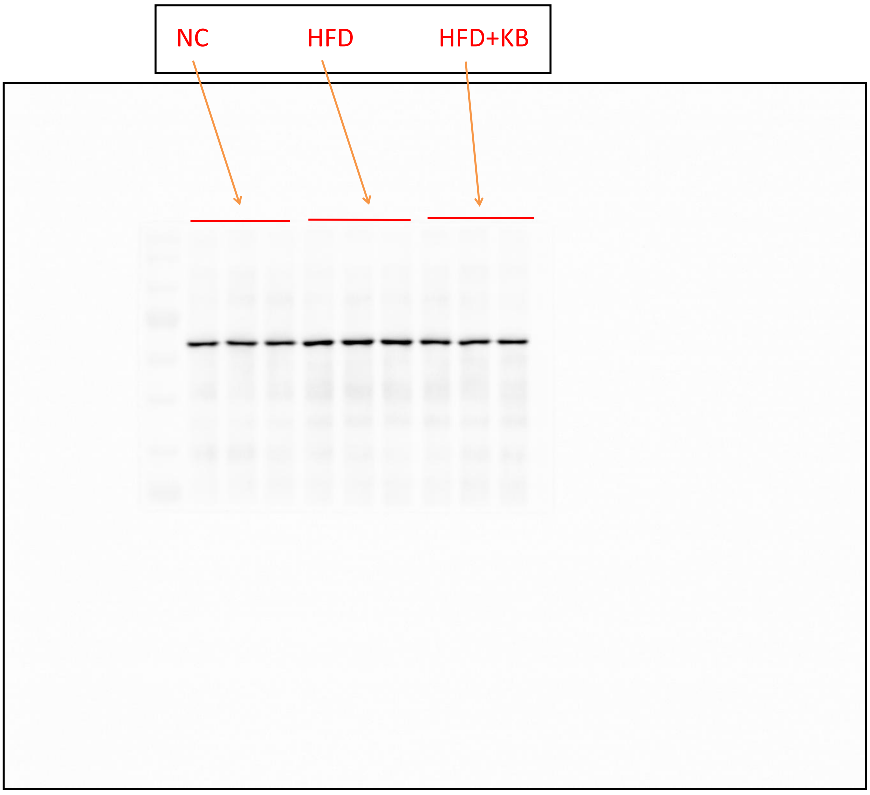

p65(65kDa) Merged image

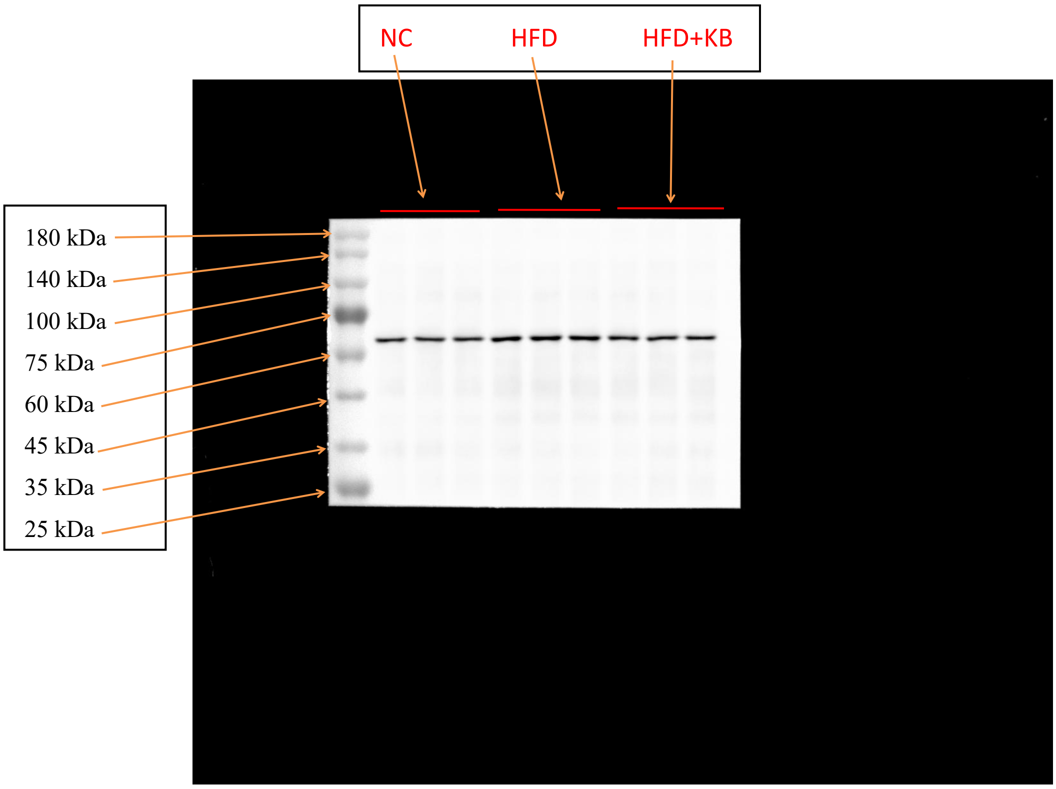

p-p65 Bright-field image

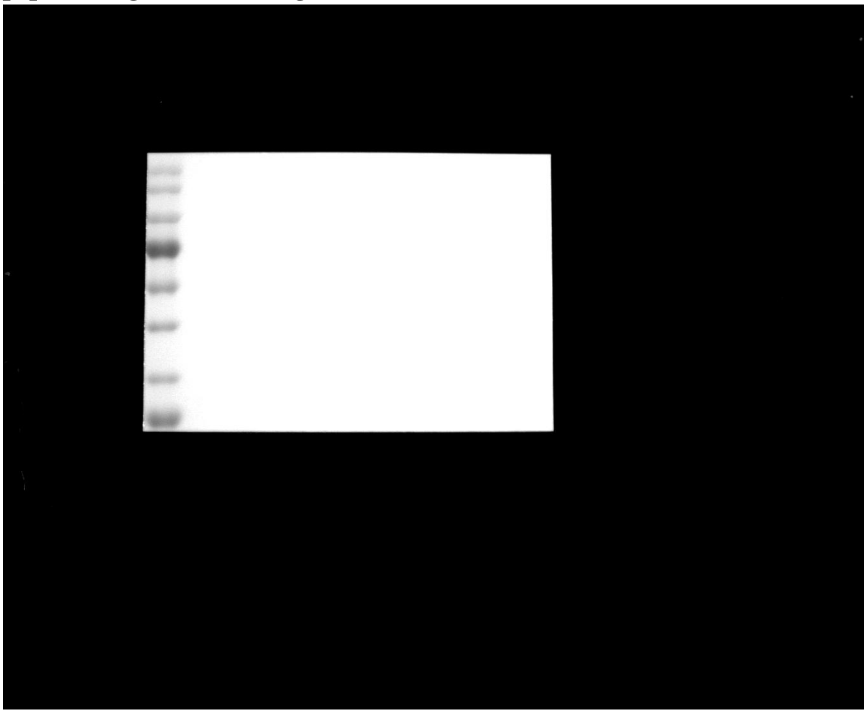

p-p65 Chemiluminescence image

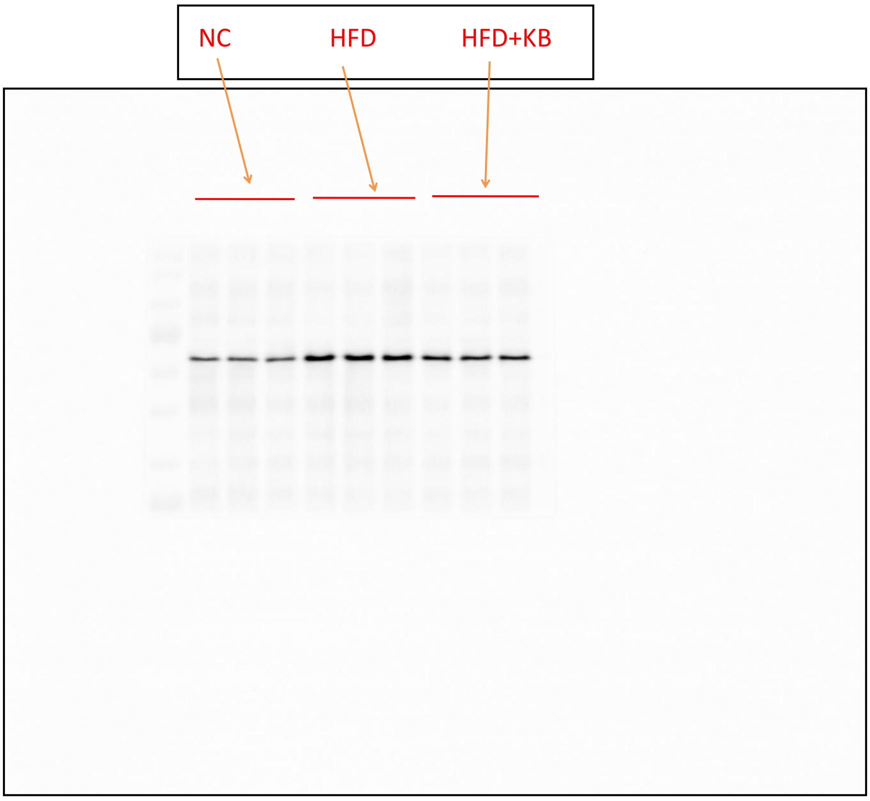

p-p65(65kDa) Merged image

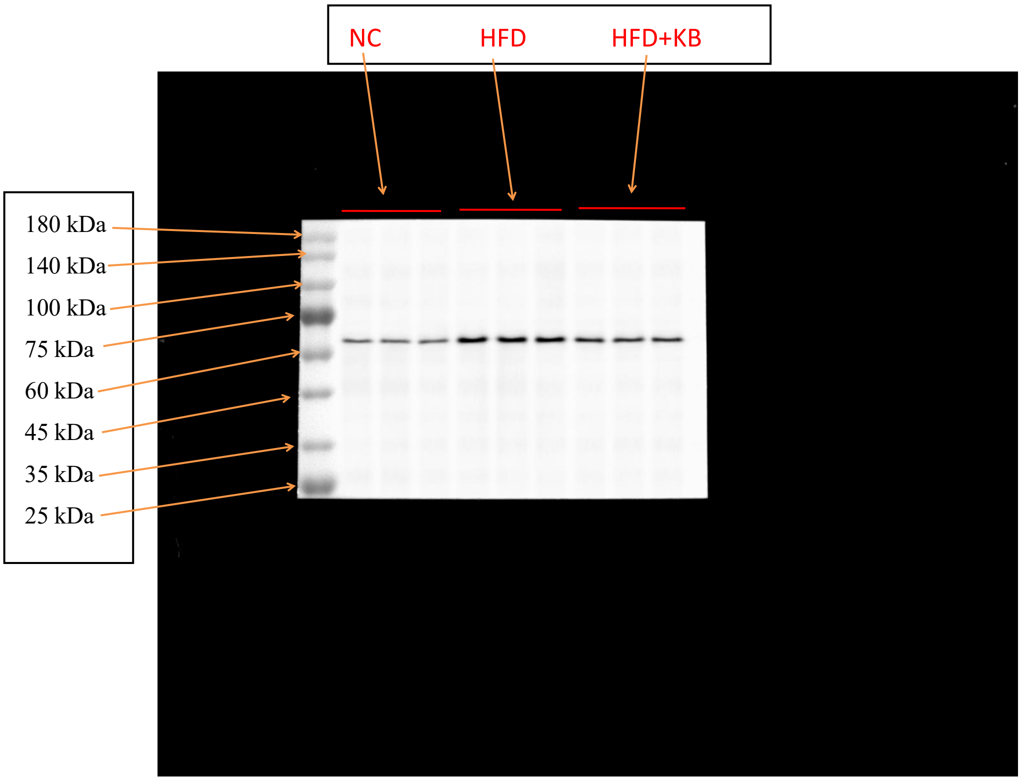

α-tubulin Bright-field image

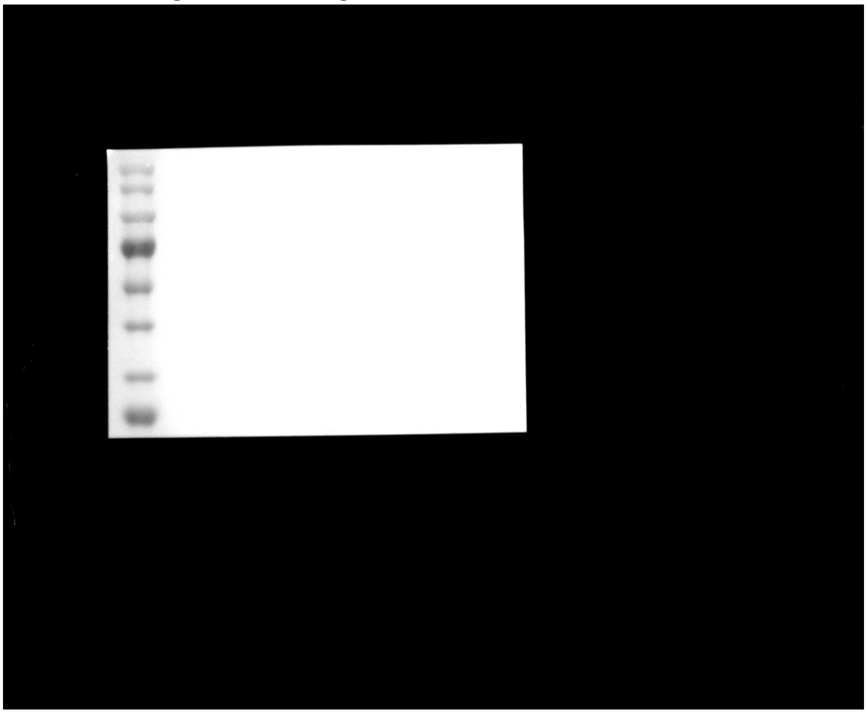

α-tubulin Chemiluminescence image

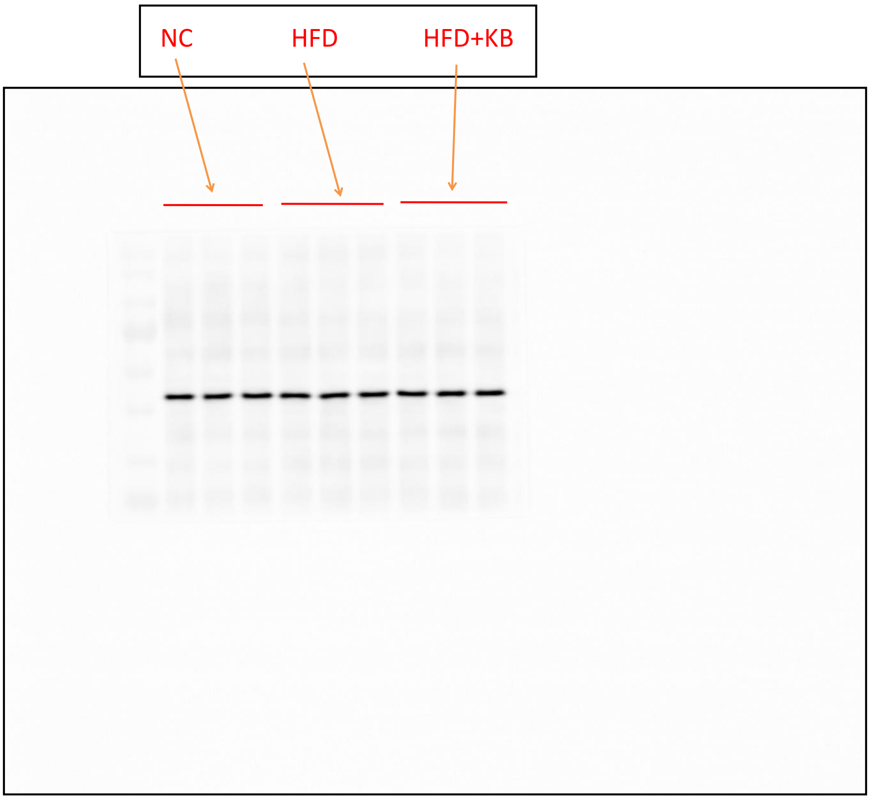

α-tubulin(50kDa) Merged image

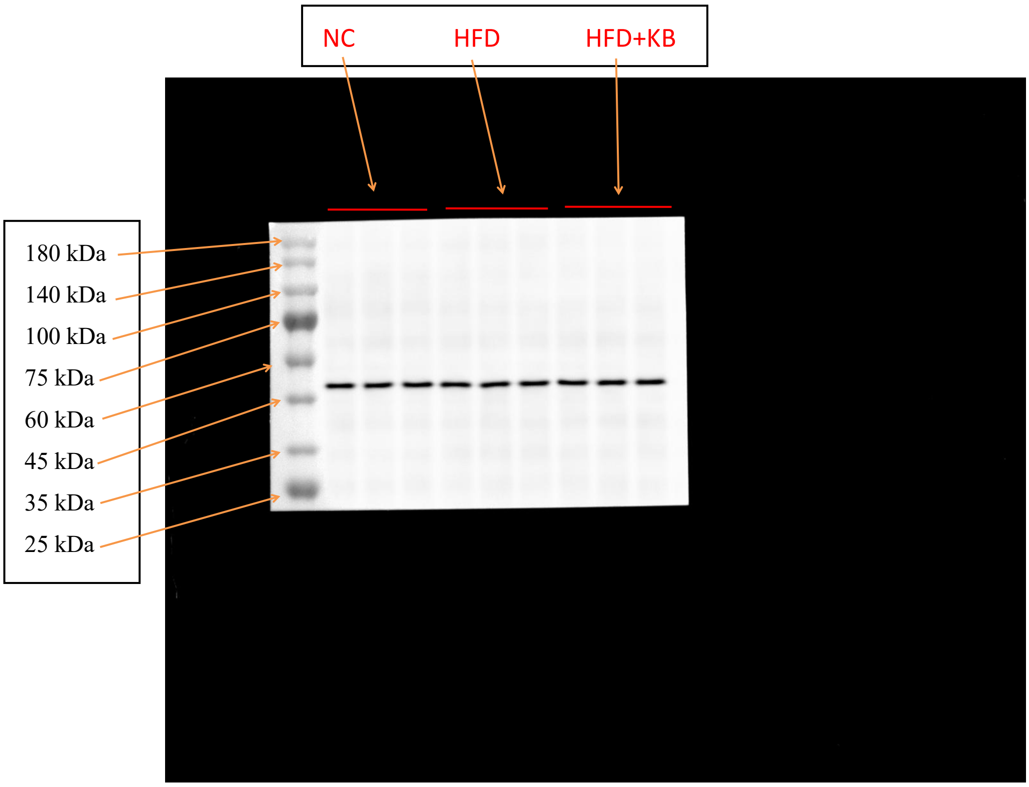

TLR4 image

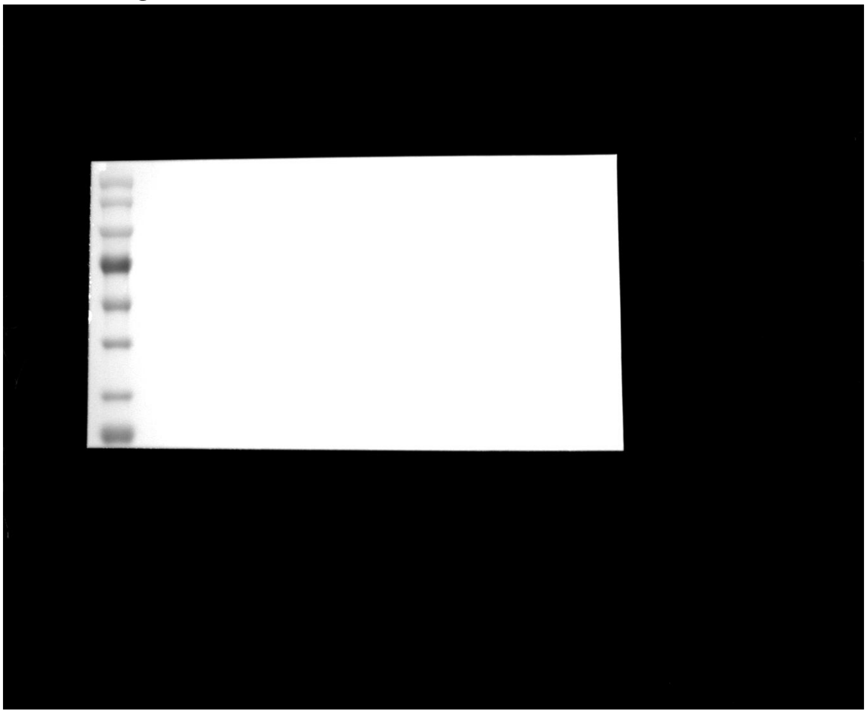

TLR4 Chemiluminescence image

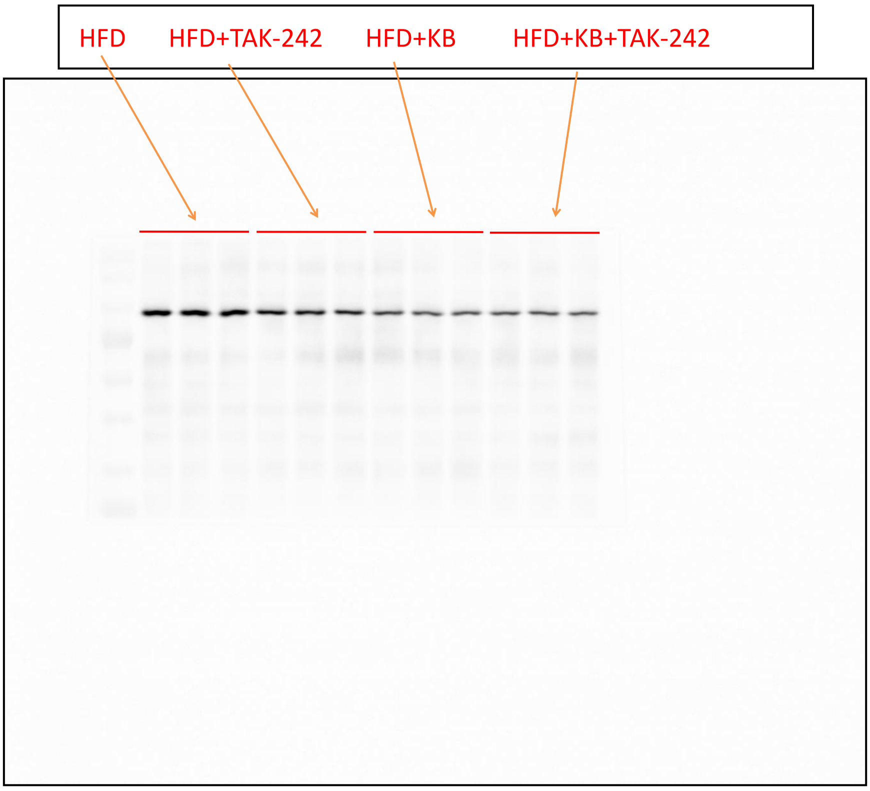

TLR4(96kDa) Merged image

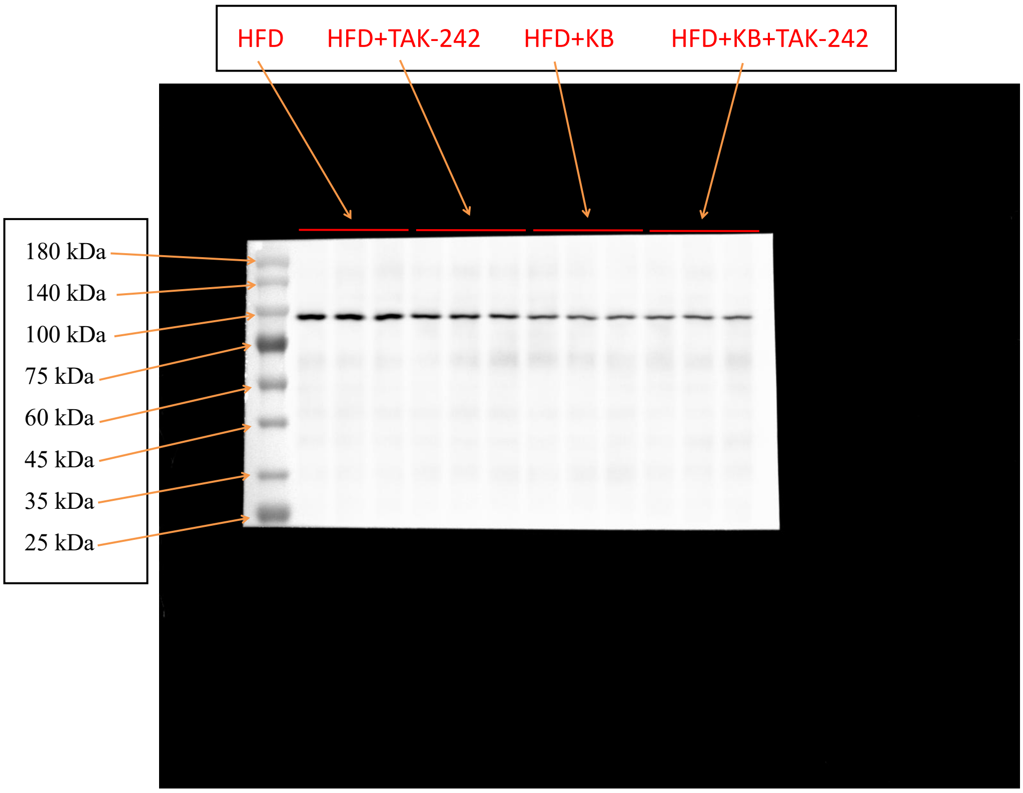

p65 Bright-field image

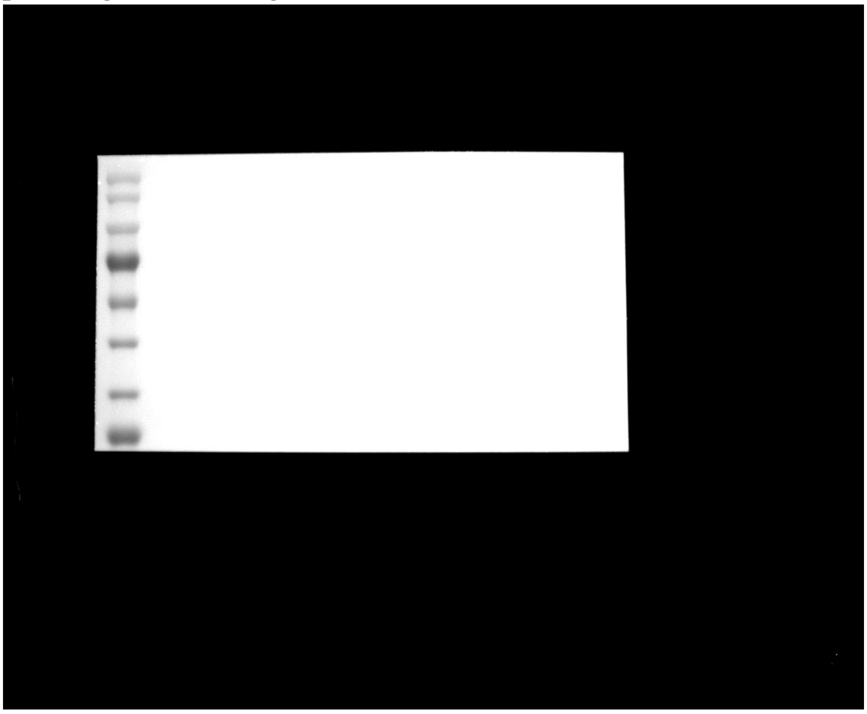

p65 Chemiluminescence image

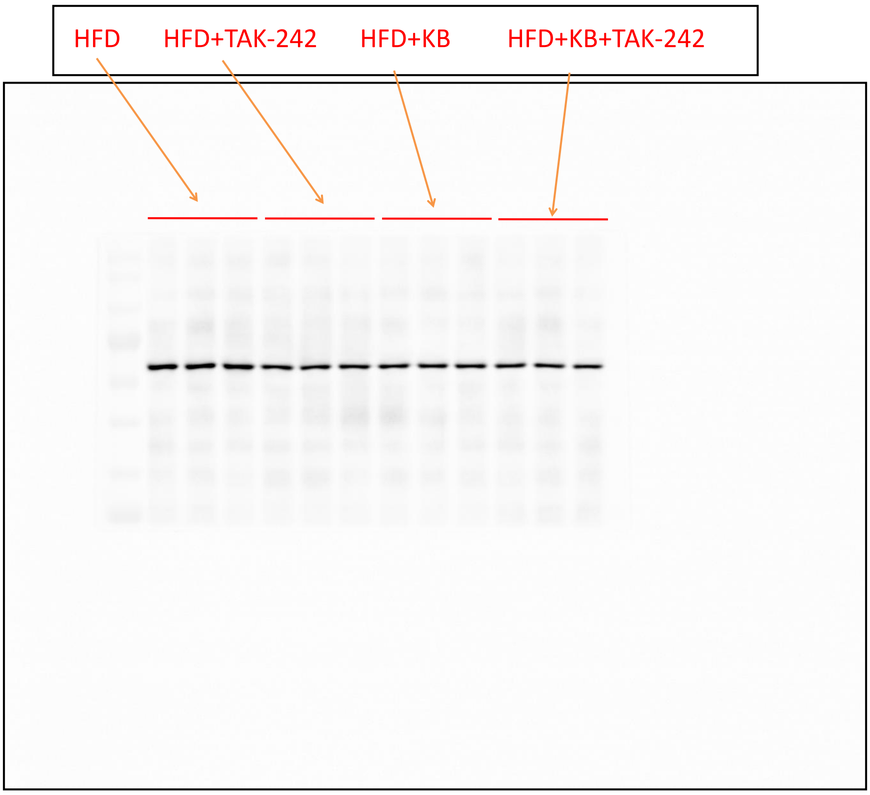

p65 (65kDa) Merged image

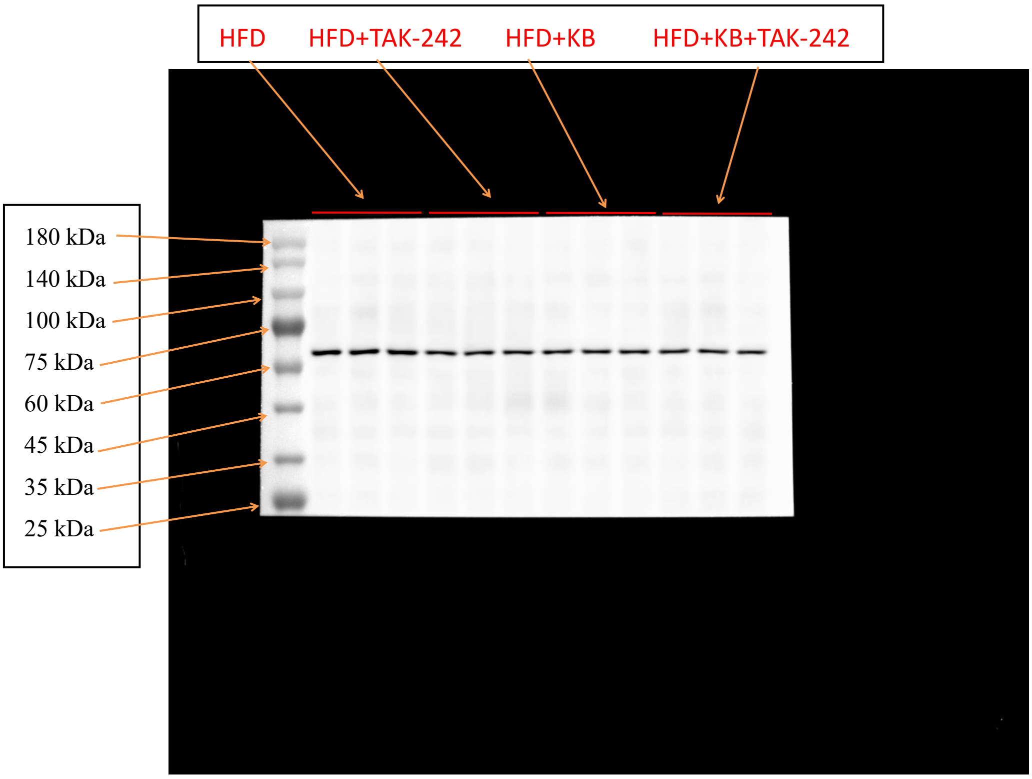

p-p65 Bright-field image

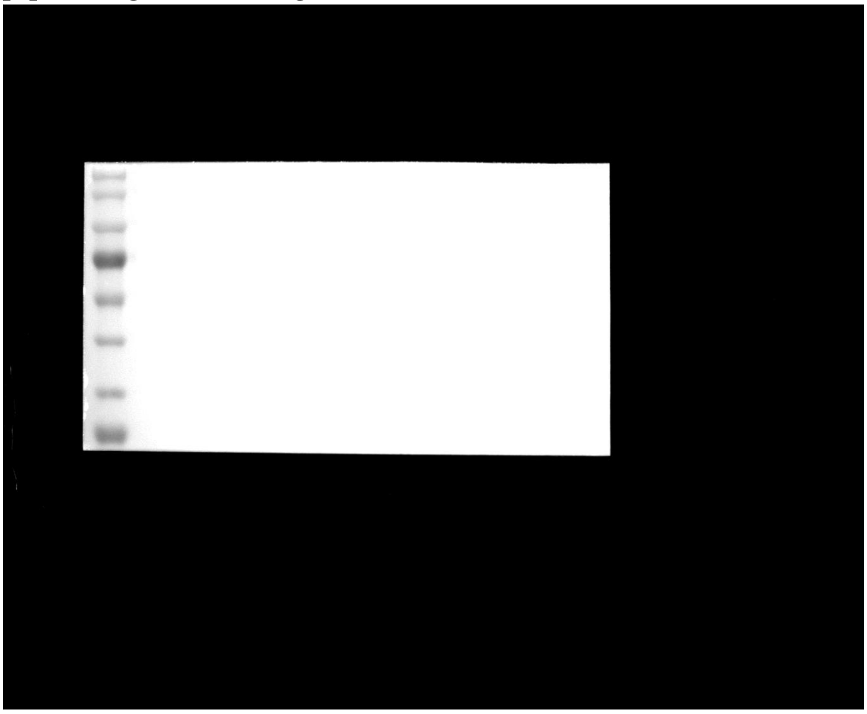

p-p65 Chemiluminescence image

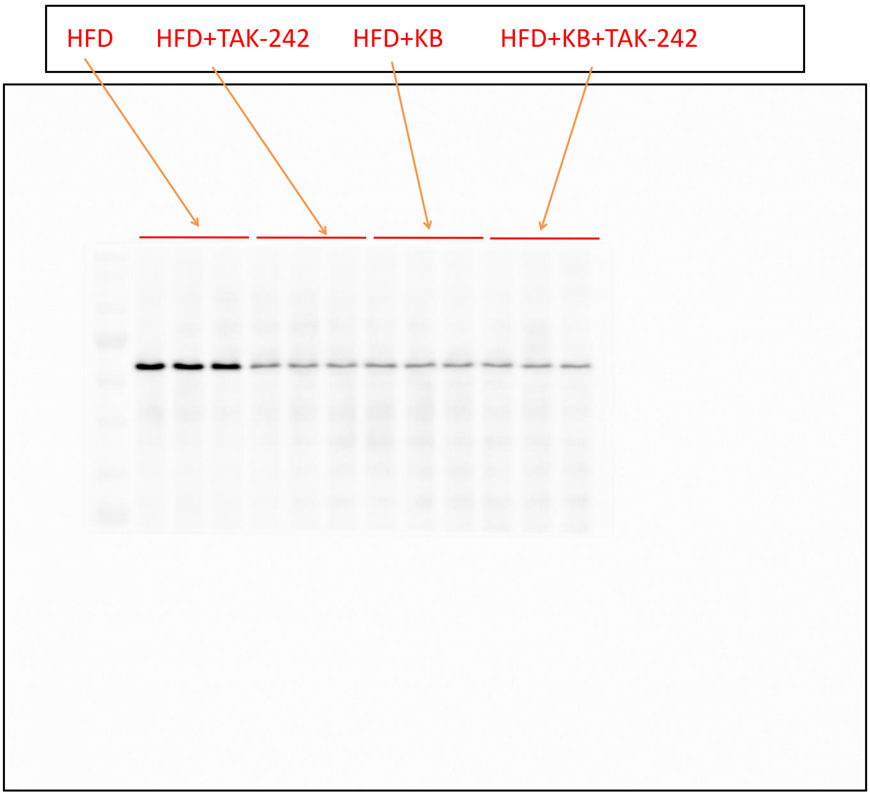

p-p65 (65kDa) Merged image

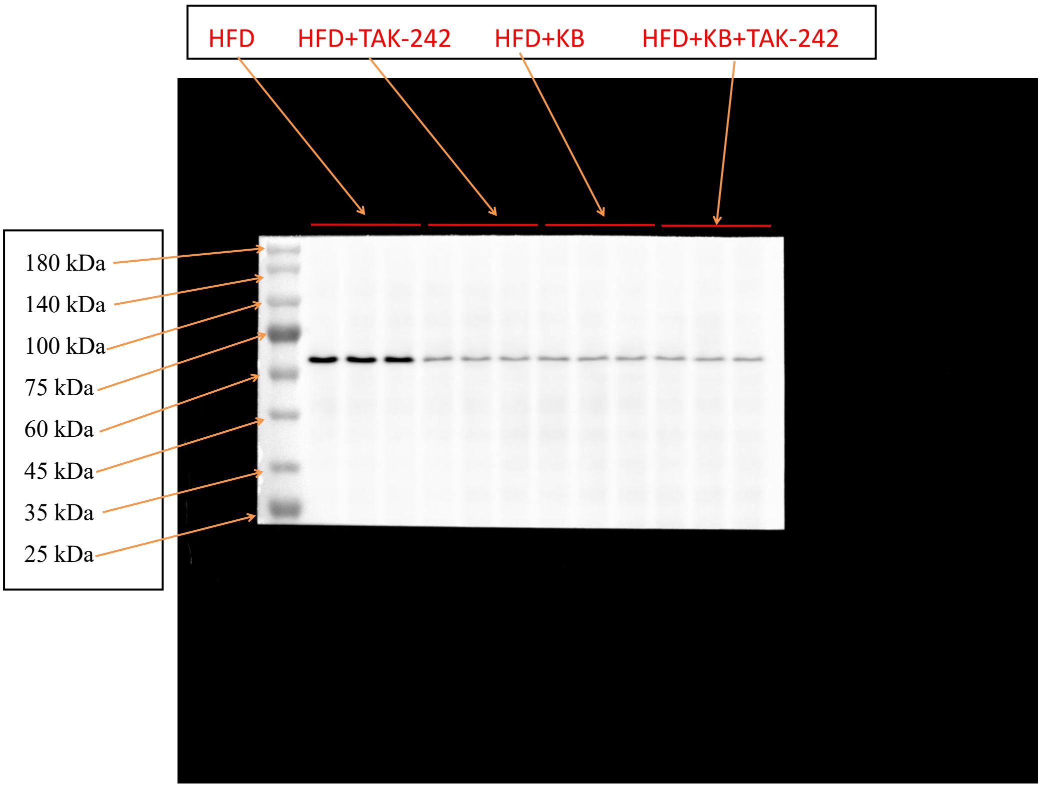

α-tubulin Bright-field image

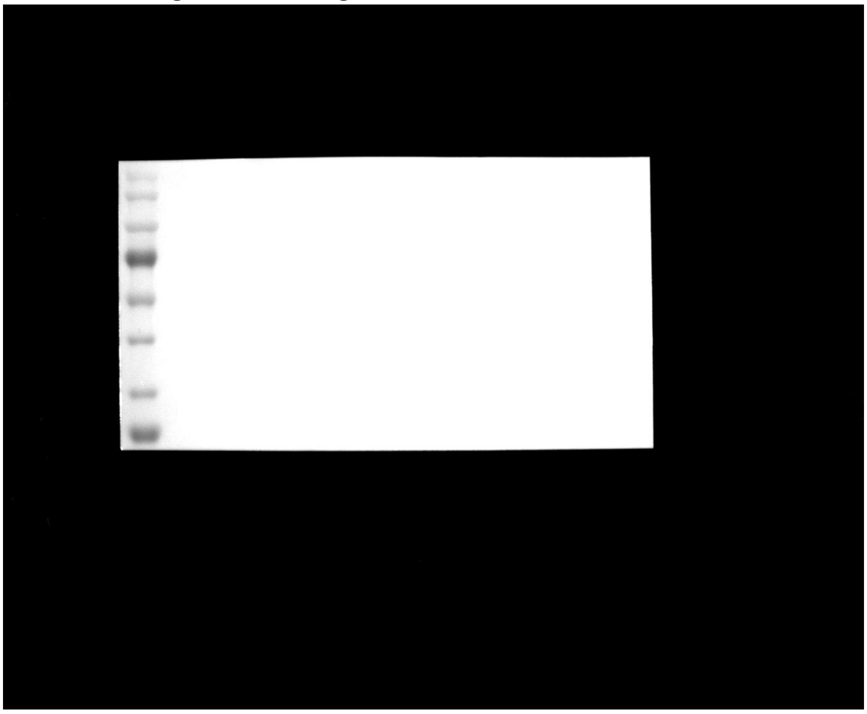

α-tubulin　Chemiluminescence image

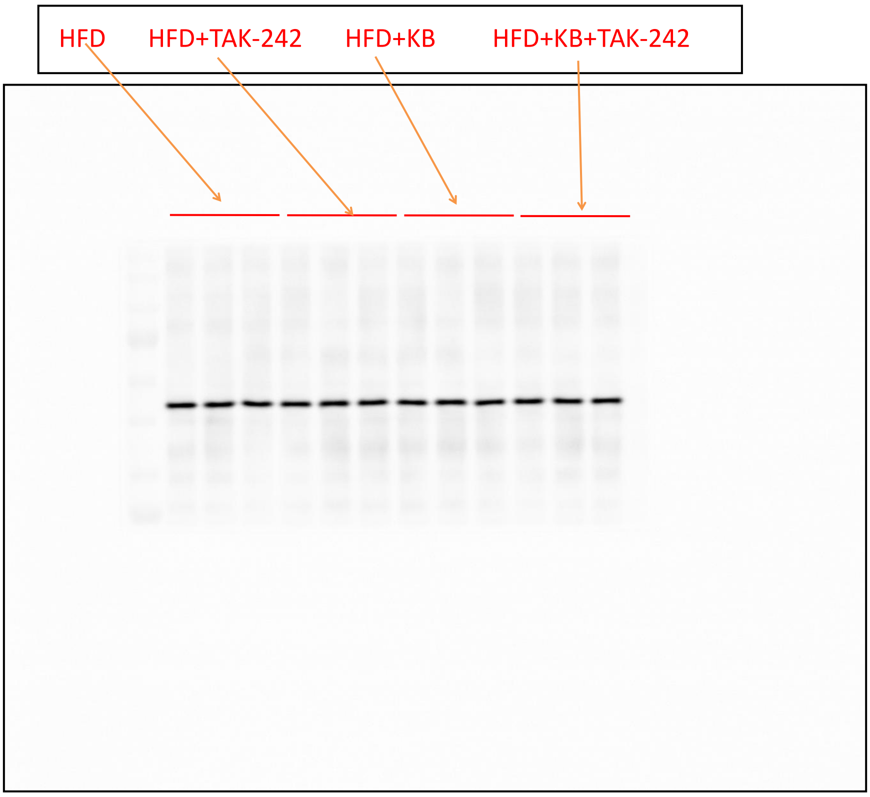

α-tubulin　(50kDa) Merged image

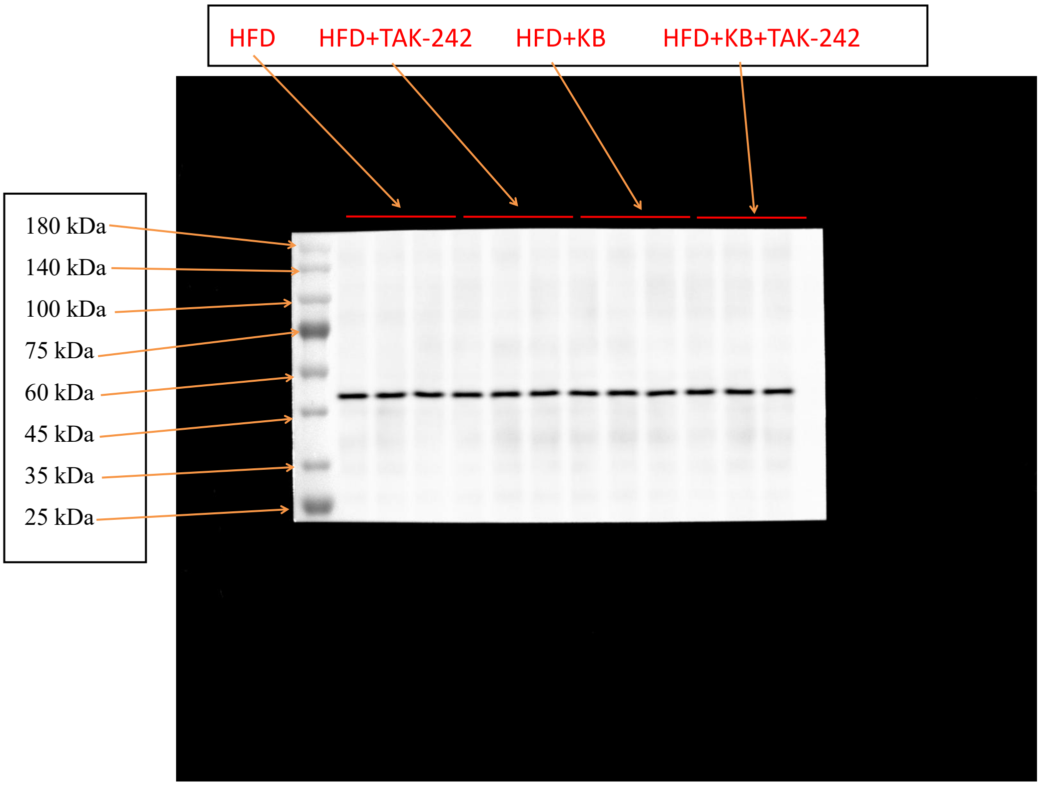

Supplement: Supplementary file 1 [file biomolecules-16-01275-s001.zip › biomolecules-4485461-original-images-1.pdf]
